# Three divergent lineages within an Australian marsupial (*Petrogale penicillata*) suggest multiple major refugia for mesic taxa in southeast Australia

**DOI:** 10.1002/ece3.1009

**Published:** 2014-03-06

**Authors:** Stephanie L Hazlitt, Anne W Goldizen, James A Nicholls, Mark D B Eldridge

**Affiliations:** 1 Department of Forest Sciences, Centre for Applied Conservation Research, University of British Columbia2424 Main Mall, Vancouver, British Columbia, V6T 1Z4, Canada; 2 School of Biological Sciences, University of QueenslandSt Lucia, Queensland, 4072, Australia; 3 Institute of Evolutionary Biology, University of EdinburghEdinburgh, EH9 3JT, U.K; 4 Australian Museum Research Institute, Australian Museum6 College St, Sydney, New South Wales, 2010, Australia; 5 Department of Biological Sciences, Macquarie UniversitySydney, New South Wales, 2109, Australia

**Keywords:** Great Dividing Range, Hunter Valley, phylogeography, rock-wallaby, southeast Australia

## Abstract

Mesic southeastern Australia represents the continent's ancestral biome and is highly biodiverse, yet its phylogeographic history remains poorly understood. Here, we examine mitochondrial DNA (mtDNA) control region and microsatellite diversity in the brush-tailed rock-wallaby (*Petrogale penicillata*;*n *=* *279 from 31 sites), to assess historic evolutionary and biogeographic processes in southeastern Australia. Our results (mtDNA, microsatellites) confirmed three geographically discrete and genetically divergent lineages within brush-tailed rock-wallabies, whose divergence appears to date to the mid-Pleistocene. These three lineages had been hypothesized previously but data were limited. While the Northern and Central lineages were separated by a known biogeographic barrier (Hunter Valley), the boundary between the Central and Southern lineages was not. We propose that during particularly cool glacial cycles, the high peaks of the Great Dividing Range and the narrow adjacent coastal plain resulted in a more significant north–south barrier for mesic taxa in southeastern Australia than has been previously appreciated. Similarly, located phylogeographic breaks in codistributed species highlight the importance of these regions in shaping the distribution of biodiversity in southeastern Australia and suggest the existence of three major refuge areas during the Pleistocene. Substructuring within the northern lineage also suggests the occurrence of multiple local refugia during some glacial cycles. Within the three major lineages, most brush-tailed rock-wallaby populations were locally highly structured, indicating limited dispersal by both sexes. The three identified lineages represent evolutionarily significant units and should be managed to maximize the retention of genetic diversity within this threatened species.

## Introduction

The recent explosion in phylogeographic studies is rapidly increasing our understanding of how different species in various habitats responded to the climatic cycles of the Plio–Pleistocene (reviewed by Avise 2000; Hewitt 2004; Knowles 2009; Shafer et al. 2009). Many taxa were isolated within refugia during glacial maxima and then spread back out as climatic conditions ameliorated; these processes resulted in characteristic genetic signals (Avise 2000; Hewitt 2000, 2011). While there has been extensive research on phylogeographic patterns in the Northern Hemisphere, patterns in the Southern Hemisphere are much less well studied (Beheregaray 2008).

In Australia, the phylogeography of the ancestral mesic biome, now confined to the coastal east, southeast, and far southwest of the continent (Byrne et al. 2011), has only been well studied for wet forest taxa from the wet tropics of northeast Queensland (reviewed by Moritz et al. 2000). Southeastern Australia, although largely dominated by drier sclerophylous vegetation, was originally identified by Keast (1961) as a major mesic refugium within Australia. Despite this region's high biodiversity, it has been relatively poorly studied (reviewed in Byrne et al. 2011), although additional data are now rapidly accumulating (e.g., Chapple et al. 2011a,2011b; Pavlova et al. 2013). In southern Australia, Pleistocene climatic cycling alternated between warm/wet during interglacial periods and colder/dryer during glacial periods, with these cycles superimposed over a more general trend of aridification (reviewed by Byrne et al. 2008). This climatic cycling is hypothesized to have caused cyclical population expansion and contraction, and some phylogeographic studies of southeastern Australian taxa show evidence of geographically discreet divergent lineages, dating from the Pleistocene or earlier (reviewed in Byrne et al. 2011), as predicted by the refugia hypothesis. However, phylogeographic studies of other southeastern taxa show a lack of genetic structure and/or little evidence of recent expansion (reviewed in Byrne et al. 2011).

Although many southeastern Australian mesic zone species are likely to have been impacted by Pleistocene climatic oscillations, sufficient data have not yet accumulated to allow general patterns to be determined (Byrne et al. 2011). Mesic species may have contracted to several major refugia as seen during glacial periods in the Northern Hemisphere (Hewitt 2000) or may have existed within multiple localized refugia (varying with species and habitat) – the so called “refugia within refugia” pattern identified for semi-arid/arid Australia (Byrne et al. 2008) and elsewhere (e.g., Shafer et al. 2009). Although past refugia are often delineated by biogeographic barriers and southeastern Australia is the most topographically complex part of the continent, few biogeographic barriers are known from the region (Schodde and Mason 1999), and the extent of their role in promoting divergence remains unclear (Byrne et al. 2011).

**Figure 1:**
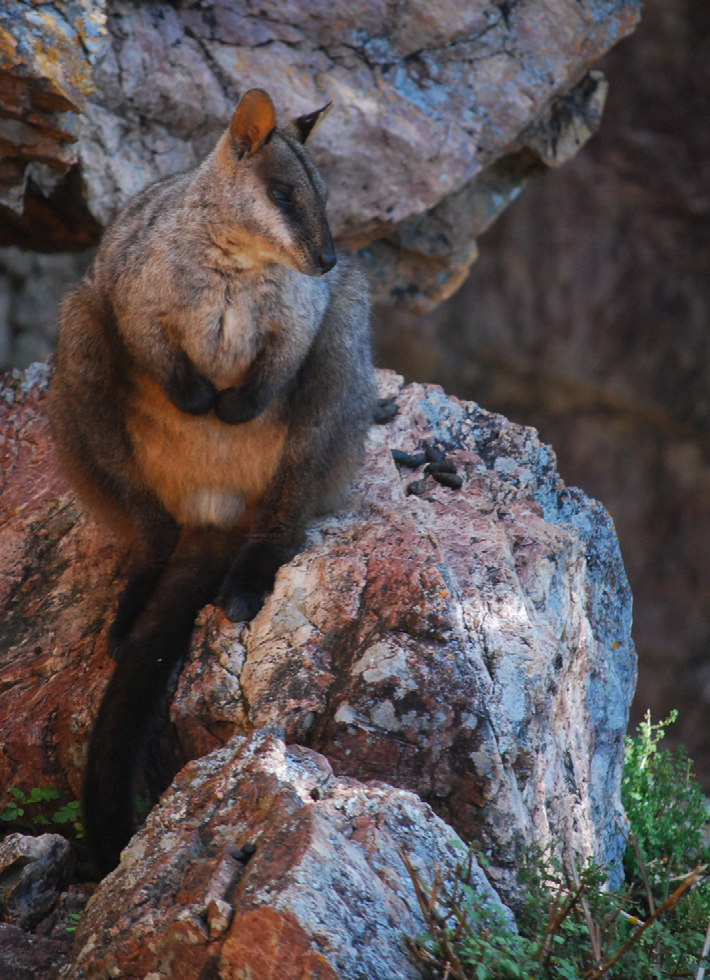
An adult male brush-tailed rock-wallaby (*Petrogale penicillata*). Photo by Katherine Tuft.

The brush-tailed rock-wallaby (*Petrogale penicillata*; Fig. 1) is an ideal species for investigating the phylogeography of southeastern Australia further. Originally distributed throughout southeastern Australia from southeast Queensland (Qld) south to Victoria (Vic) and west into the New South Wales (NSW) semi-arid zone, the brush-tailed rock-wallaby occupies a range of habitats, although most typically the poorly studied drier sclerophyll forests and woodlands (Eldridge and Close 2008). Rock-wallabies' reliance on rocky habitat (Eldridge 2008) and their naturally limited dispersal abilities (Eldridge et al. 2010) make them sensitive indicators of historic disruption to gene flow and therefore useful indicators of biogeographic barriers (Potter et al. 2012b). Previous population genetic studies of brush-tailed rock-wallabies have shown evidence of significant local and regional genetic structuring, although these studies were all based on limited sampling or geographic coverage (Browning et al. 2001; Eldridge et al. 2001a; Hazlitt et al. 2006, 2010; Paplinska et al. 2011). Browning et al. (2001) proposed that the brush-tailed rock-wallaby contained three divergent mtDNA lineages (potential evolutionarily significant units; ESUs), but their sampling throughout the species range was limited. Paplinska et al. (2011) explored the distribution of the three “putative ESUs” using published data and new samples extracted from museum specimens. Their data supported the existence of three divergent mtDNA lineages but involved analysis of only a short fragment (<200 bp) of the control region. Thus, a more comprehensive analysis using both mtDNA and nuclear markers is required to confirm the three ESU hypothesis.

The brush-tailed rock-wallaby is the southernmost in a chain of recently derived rock-wallaby species (Campeau-Peloquin et al. 2001; Potter et al. 2012a) distributed parapatrically down the ranges of eastern Australia. These species show evidence of the impact of Pleistocene cycling on speciation (Potter et al. 2012a), but intraspecific population structuring has only been explored in *P. penicillata*. At the northern extremity of its range, the brush-tailed rock-wallaby contacts its putative sister species Herbert's rock-wallaby (*Petrogale herberti*) at a narrow hybrid zone (Eldridge and Close 2008). Brush-tailed rock-wallabies have declined significantly in the last 150 years, with many local population extinctions, especially in the south and west of their range (Eldridge and Close 2008). They are now listed as “Vulnerable” nationally (“Endangered” in NSW, Critically Endangered' in Vic) and are actively managed especially in NSW and Vic.

We aimed to sample the brush-tailed rock-wallaby across its range and use both mitochondrial DNA (mtDNA) and microsatellite markers to: (1) determine phylogeographic patterns of population differentiation; (2) infer the biogeographic barriers associated with these patterns; (3) estimate the timing of these divergences; (4) use these findings to increase our understanding of the phylogeographic processes that have shaped the distribution of diversity in southeastern Australia; and (5) assess the implications of these spatial patterns for the management of the species.

## Methods

### Sample collection and molecular methods

Tissue samples were collected from 1982 to 2004, from 31 locations throughout the species' range in southeastern Australia (*n* = 279; Table 1, Fig. 1), by live-trapping (e.g., Browning et al. 2001; Eldridge et al. 2004; Hazlitt et al. 2006) and opportunistically (e.g., road kill). Total cellular DNA was extracted from frozen and alcohol-preserved tissue using standard techniques (Sambrook et al. 1989). Samples were genotyped using 11 polymorphic microsatellite loci. Six loci were derived from the allied rock-wallaby (*P. assimilis*: Pa55, Pa297, Pa385, Pa593, Pa595, Pa597; Spencer et al. 1995) and five from the tammar wallaby (*Macropus eugenii:* Me2, Me14, Me15, Me16, Me17; Taylor and Cooper 1998). Individual genotypes were detected using either *α*^33^-P labeling visualized by autoradiography (described in Spencer et al. 1995; Taylor and Cooper 1998) or using fluorescent labeling resolved using an automated Amersham Biosciences MegaBACE 500 capillary sequencer (described in Hazlitt et al. 2006). Fifteen to 35 individuals per locus were scored using both methods to ensure consistency, with an overall genotyping scoring error of 1.3% estimated between methods (further details described in Hazlitt et al. 2006).

**Table 1:** Brush-tailed rock-wallaby sample sites, sample sizes, numbers of individuals sequenced for the control region and numbers of mitochondrial DNA haplotypes detected. Site numbers correspond to those in Figure 1.

Site No	Site (Abbreviation)	*n*	n (mtDNA)	Number of haplotypes	GenBank Accession
	Queensland					
1	Yarraman Creek (YC-Q)	1	1+	1	AY040890
2	Cooyar Creek (CC-Q)	4	4	1	AY040890
3	Nukinenda Falls (NF-Q)	1	1+	1	AY040889
4	Sommerset Dam (SD-Q)	1	1+	1	AY040890
5	Crows Nest NP (CN-Q)	12	12	2	KJ396276, AY040890
6	Perseverance Dam (PD-Q)	16	11	2	KJ396276, KJ396277
7	Emu Creek (EC-Q)	10	7	2	EU887006, EU887009
8	Farm Creek (FC-Q)	11	9	1	EU887005
9	Farm Creek East (FCE-Q)	10	4	3	EU887005, EU887010, EU887011
10	Hurdle Creek (HC-Q)	54	47	5	EU887004-EU887008
	New South Wales					
11	Bonalbo (Bon-N)	1	1+	1	AF357277
12	Armidale (Arm-N)	12	12	2	AF357279, AY040887
13	Warrumbungles (War-N)	1	1+	1	AY040884
14	Woko National Park-2 (Wo2-N)	2	2^b^	1	KJ396285
15	Woko National Park-1 (Wo1-N)	2	2^b^	1	KJ396284
16	Martindale (Mar-N)	7	7	2	KJ396280, KJ396281
17	Yellow Rock (YR-N)	1	1+	1	KJ396283
18	Drews Creek (DC-N)	20	20	1	AF357281
19	Ingles Road (IR-N)	29	28	1	AF357282
20	Bowmans Road (BR-N)	20	20	1	AF357282
21	St Albans (StA-N)	8	8	3	KJ396278, KJ396279, KJ396282
22	Winmalee (Win-N)	1	1+	1	AY040886
23	Jenolan Caves (Jen-N)	30	30	1	AF348699
24	Taralga (Tar-N)	2	2*	1	AF357280
25	Kangaroo Valley (KV-N)	4	4	1	AF357278
	Victoria					
26	Rocky Plains Creek (RPC-V)	8	8	1	AF357272
27	Little River Gorge (LRG-V)	4	3^a^	2	AF3572723, AF357276
28	Farm Creek (FC-V)	2	2^a^	1	AF3572723
29	Gelantipy Creek (GC-V)	2	2^a^	1	AF357275
30	Currie Creek (CC-V)	1	1+	1	AF357274
31	Grampians (Gra-V)	2	2*	1	AF357271

* Sample size=census size;

+ ,not included in AMOVA;

a pooled for AMOVA, estimates of diversity and differentiation (designated Mer-Vic);

b pooled for AMOVA.

Mitochondrial DNA control region (*CR*) was amplified using conserved marsupial primers (L15999M and H16498M Fumagalli et al. 1997), and individuals assigned to haplotypes using SSCP (as previously described Sunnucks et al. 2000; Eldridge et al. 2001a) (Table 1). Sequence data were obtained for each unique haplotype using BigDye termination chemistry and resolved using automated capillary sequencers. Over 500 base pairs (bp) of *CR* sequence was obtained from 3 to 11 individuals of each SSCP haplotype except where a unique haplotype was identified in only one or two individuals. Some mtDNA *CR* data were also available from previous studies (see Table 1 for GenBank Accession Numbers). In addition, sequences from four other rock-wallaby species were included: two from *P. herberti* the putative sister species (Potter et al. 2012a)(AF357284 and AY040892), one from *P. assimilis* (a northerly species from the same species complex), and one sequence from each of *P. lateralis* (AF348694) and *P. purpureicollis* (AY057377) for use as outgroups.

### Population genetic structure inferred from mtDNA

The program MEGA v5 (Tamura et al. 2007) was used to check sequences and create an initial alignment (using default parameters in ClustalW), which was then adjusted by eye. Phylogenetic relationships among unique *CR* haplotypes were reconstructed using Bayesian methods implemented in the program MrBayes v3.1.2 (Ronquist and Huelsenbeck 2003). For this analysis, a GTR+I+G model was selected using Modeltest 3.06 (Posada and Crandall 1998). Indels were included as a second data partition within the same analysis, coded as binary data with a variable ascertainment bias. Rate variation within this second partition was initially modeled using a gamma distribution, but an examination of the posterior distribution indicated the data were not informative with respect to this parameter, so in the final model, rates for this data partition were set to equal. In all MrBayes analyses, four chains per run and two independent runs were used. A temperature setting of 0.2 and run length of 6,000,000 generations allowed adequate mixing among chains and convergence between runs. Parameters and trees were sampled every 1000 generations with tree topology and node support assessed over the final 500,000 generations. Convergence between runs, convergence of parameters, and appropriate levels of chain swapping were assessed using Tracer v1.4 (Rambaut and Drummond 2007).

We used Bayes factors, estimated using twice the difference in the natural log of the harmonic mean of model likelihoods of each model (2ΔlnHML), assessed following Kass and Raftery (1995), to assess the applicability of a molecular clock. Models run under the three strict clock models implemented in MrBayes (uniform, birth-death and coalescent) were compared with a nonclock model. In all cases, the data strongly supported the use of a molecular clock (all 2ΔlnHML > 47.2). The software BEAST v1.4.8 (Drummond and Rambaut 2007) was used to assess node ages, using only the substitution data matrix. The topology of the haplotype tree, with multiple clusters of closely related sequences at the ends of long branches, suggested that these data are at the interface between data best-modeled using coalescent demographic processes and those using speciation processes. Applying a single demographic model across the whole tree would have been inappropriate, as each cluster represented an independently evolving unit, and similarly, models which only incorporate lineage speciation/extinction rates would have been inappropriate at shallower levels in the tree. Hence, we analyzed these data using a Bayesian skyline coalescent model, allowing population size to fluctuate over the tree, therefore eliminating the constraints any particular demographic/speciation model would impose. Bayes factor comparison supported a relaxed lognormal over a strict clock (2ΔlnHML = 26.96). All individuals, rather than just a single representative of each haplotype, were entered into the BEAST analyses to allow a more realistic approximation of the coalescent model of sequence divergence and hence lineage divergence times. Two independent runs of each model, each of 12,000,000 generations sampled every 2000 generations, were performed with parameter estimates based on the final 2,000,000 generations. Because estimates of a clock rate for mammalian *CR* vary widely (6–38% pairwise divergence per million years: Troy et al. 2001; Savolainen et al. 2004; Saarma et al. 2007), we used a mid-range rate of 15% pairwise divergence per million years (Birungi and Arctander 2000); however, given this variation, the resulting time estimates should be treated with caution. The median age and 95% highest posterior probability bounds for major nodes were calculated in Tracer v1.4 and then mapped onto the MrBayes *CR* haplotype consensus tree.

The hypothesis that three differentiated geographic groups exist within brush-tailed rock-wallabies was tested using the hierarchical analysis of genetic differentiation in ARLEQUIN 3.1 (AMOVA Excoffier et al. 2005). Population pairwise Φ_*ST*_ was calculated using the HKY+G distance model, described below, with significance evaluated with 10,100 permutations. Few haplotypes were shared among populations; therefore, standard *F*_*ST*_ values (based only on haplotype frequency) would have been unlikely to provide reliable estimates of genetic differentiation. Some of the 31 populations were excluded or pooled with neighboring sites for the AMOVA, as the Φ_*ST*_ distance estimates derived from “populations” with very small sample sizes of only one or two samples were likely to be poor estimates of the true values. Populations with sample sizes less than four were excluded (Yarraman Creek, Warrumbungles, Winmalee, Yellow Rock, Bonalbo, Sommerset Dam, Nukinenda Falls, Currie Creek) unless the small sample size was reflective of the census size (Grampians, Taralga). Populations with *n* < 4 but within 5 km or less of a neighboring site were pooled (Woko National Park sites 1 and 2, Farm Creek – VIC, Little River Gorge and Gelantipy Creek), resulting in 20 populations (Table 1).

Isolation by distance (IBD) was tested across the entire range and within two of the identified phylogeographic groups (Northern and Central lineage). Sampling for the Southern lineage was not adequate for an IBD analysis. We compared geographic distances (km) and corrected average pairwise population differences, calculated in ARLEQUIN, using Mantel tests with 10,000 permutations in GenAlEx v6.1 (Peakall and Smouse 2006). Pairwise differences were based on a HKY (Tamura in ARLEQUIN) model of haplotype distance with a gamma distribution value of 0.136 (accounting for +G, determined in MODELTEST). Similar results were obtained if alternative distance models were used. Evidence for historic demographic expansion events was tested by mismatch analysis (sum of squared deviations (SSD) and Harpending's raggedness index (R)), as well as Tajimas's D and Fu's Fs tests of neutrality in ARLEQUIN, examining all haplotypes from across the species' range and within each of the three major lineages (Southern, Central, Northern).

### Population genetic structure inferred from microsatellites

Microsatellite analyses were conducted using data from 14 sites (*n* = 247; Table 2). These data included 13 colonies (individuals inhabiting a discreet habitat patch) with sample sizes of *n* ≥ 7 (Table 2), and three colonies with smaller sample sizes (*n* ≤ 4) that were merged together to create a single population because they were within a 5 km distance of each other (Little River Gorge, Gelantipy Creek, and Farm Creek, Victoria, *n* = 8; Table 2), as dispersal has been detected over this distance (Eldridge et al. 2001a,2001b). Exact tests for deviations from Hardy–Weinberg equilibrium for each locus and linkage disequilibrium between loci were carried out for each population in GENEPOP 3.1 (Raymond and Rousset 1995) using the Markov chain method with 1000 iterations. When performing multiple comparisons, we adjusted the statistical significance level using the sequential Bonferroni procedure at *α *= 0.05 (Rice 1989). Observed and expected heterozygosity (*H*_*O*_ and *H*_*E*_) for all loci were estimated using the program POPGENE 1.3.2 and allelic richness (*A*_*R*_, the average number of alleles per locus standardized for unequal sample sizes between sites) was calculated for each sampled colony using FSTAT version 2.9 (Goudet 1995).

**Table 2:** Genetic diversity (mean ± SE) at 11 microsatellite loci in 14 brush-tailed rock-wallaby populations from southeastern Australia. See Supplementary Table S1 for population allele frequencies.

Site No	Site (Abbreviation)	*n*	AD (±SE)	AR (±SE)	Ho (±SE)	He (±SE)
	Queensland						
5	Crows Nest NP (CN-Q)	12	3.6 (0.53)	2.2 (0.17)	0.52 (0.06)	0.52 (0.07)
6	Perseverance Dam (PD-Q)	16	3.7 (0.41)	2.3 (0.13)	0.52 (0.07)	0.57 (0.05)
7	Emu Creek (EC-Q)	10	4.4 (0.56)	2.5 (0.14)	0.67 (0.06)	0.64 (0.05)
8	Farm Creek (FC-Q)	11	4.5 (0.43)	2.4 (0.17)	0.66 (0.07)	0.61 (0.04)
9	Farm Creek east (FCE-Q)	10	3.9 (0.56)	2.5 (0.12)	0.73 (0.09)	0.58 (0.07)
10	Hurdle Creek (HC-Q)	54	6.1 (0.61)	2.5 (0.11)	0.68 (0.05)	0.66 (0.04)
	New South Wales						
12	Armidale (Arm-N)	12	3.9 (0.37)	2.5 (0.14)	0.71 (0.07)	0.62 (0.05)
16	Martindale (Mar-N)	7	4.4 (0.41)	2.7 (0.10)	0.71 (0.05)	0.68 (0.03)
18	Drews Creek (DC-N)	20	4.3 (0.51)	2.4 (0.13)	0.64 (0.06)	0.60 (0.05)
19	Ingles Road (IR-N)	29	5.2 (0.35)	2.6 (0.08)	0.75 (0.03)	0.69 (0.02)
20	Bowmans Road (BR-N)	20	5.1 (0.42)	2.6 (0.12)	0.74 (0.06)	0.67 (0.04)
23	Jenolan Caves (Jen-N)	30	3.3 (0.33)	2.0 (0.14)	0.56 (0.08)	0.49 (0.06)
	Victoria						
26	Rocky Plains Creek (RPC-V)	8	3.3 (0.25)	1.6 (0.11)	0.43 (0.09)	0.33 (0.06)
27–29	*Little River (4), Farm Creek (2), Gelantipy Creek (2) (Mer-V)	8	2.1 (0.36)	2.3 (0.15)	0.45 (0.07)	0.54 (0.06)
	All Populations	247	13.4 (1.01)	3.2 (0.07)	0.63 (0.02)	0.59 (0.02)

Average allelic richness (AR) corrected for *n *= 7.

* Samples pooled for analyses (see methods).

We calculated pairwise values of *F*_*ST*_ for all colonies and tested for significance with FSTAT version 2.9 (Goudet 1995). Population genetic structure was also inferred using a Bayesian model-based clustering analysis in the program STRUCTURE 2.3.1 (Pritchard et al. 2000). STRUCTURE was run under the admixture model with alpha inferred from the data, allele frequencies uncorrelated and lambda set to 1.0. After a burn-in of 100,000 and 200,000, iterations were performed. For the whole data set, we tested the number of genetic clusters (populations, *K*) present using values of *K* between 1 and 14, with 10 replicates of each. The inferred number of populations within the sample was deduced using both maximum posterior probability (*L*(*K*) Pritchard et al. 2000), and maximum delta log likelihood (ΔK Evanno et al. 2005) implemented in STRUCTURE HARVESTER 0.6.93 (Earl and vonHoldt 2012). Each identified cluster was subsequently rerun to test for additional substructuring within clusters. Finally, we created an unrooted neighbor-joining tree to visualize genetic similarity among the sample sites. The unrooted neighbor-joining tree was based on the average allele sharing genetic distance (Dps) among the 14 populations. Dps values and bootstrap iterations (1000) were calculated in MICROSAT and constructed using the NEIGHBOR and CONSENSUS subroutines in PHYLIP version 3.5 (Felsenstein 1995), with the tree created in TREEVIEW version 1.5.

## Results

### Population genetic structure inferred from mtDNA

*CR* sequence was obtained for 259 individuals, resulting in an alignment of 532 bp. There were 82 variable sites (72 parsimony informative) among the 254 *P. penicillata* individuals, resulting in 36 haplotypes (Table 1). Novel haplotype sequences generated in this study are available from GenBank (Table 1).

**Figure 2:**
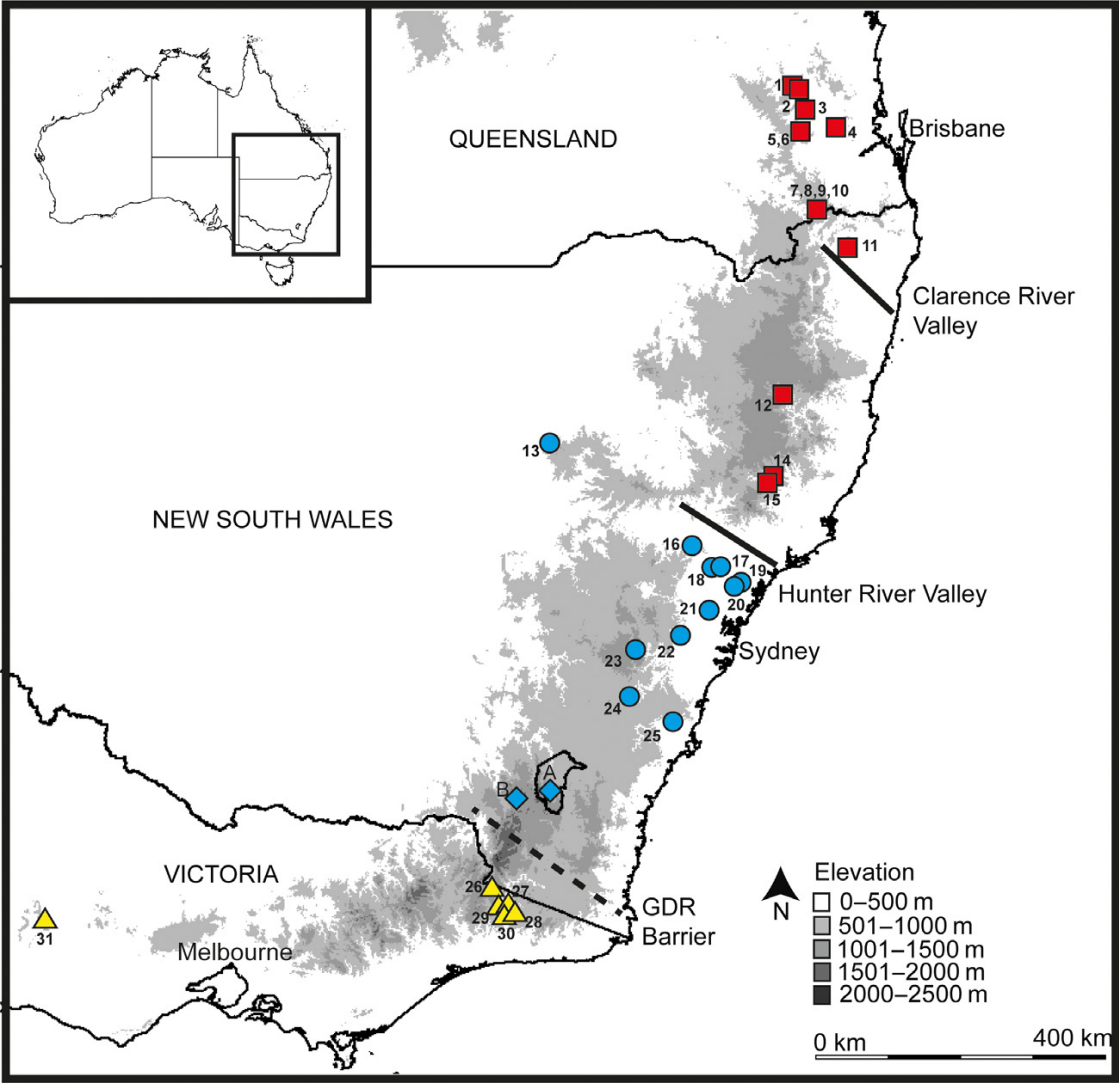
Location of brush-tailed rock-wallaby populations sampled in this study from across the species' range in southeastern Australia. The distribution of the three distinct mitochondrial lineages (see Fig. 3) is indicated by: red squares, Northern lineage; blue circles, Central lineage; and yellow triangles, Southern lineage. The blue diamonds represent the collection localities for two museum specimens assigned to the Central lineage by Paplinska et al. (2011).

**Figure 3:**
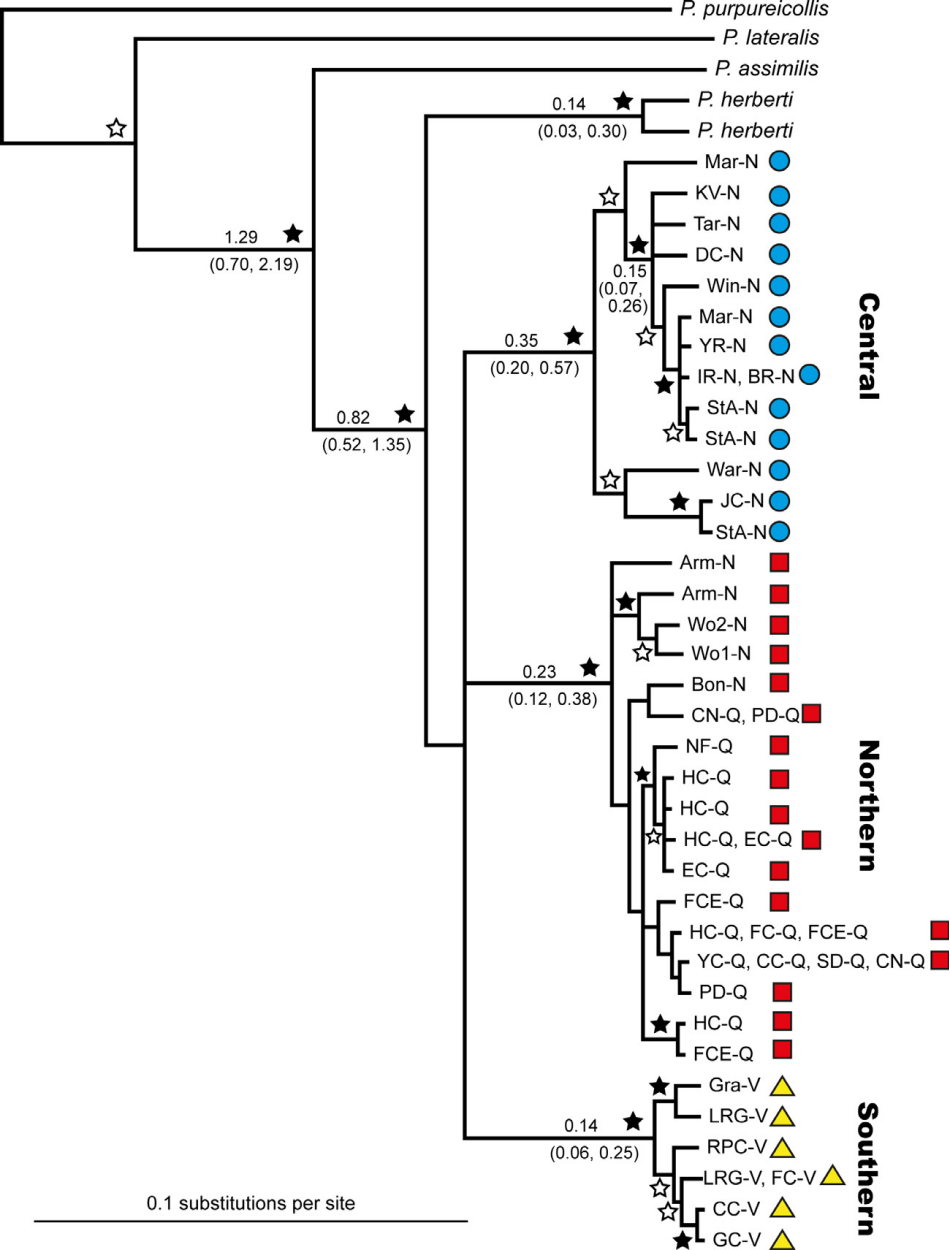
Bayesian 50% majority-rule consensus tree for the 36 brush-tailed rock-wallaby control region haplotypes obtained in this study. Black stars indicate ≥95% posterior probability node support, white stars indicate node support of ≥70%, other nodes present have posterior support of 50–69%. Numbers on branches are node ages in millions of years, with 95% highest posterior density bounds in parentheses. The three identified brush-tailed rock-wallaby mitochondrial lineages are indicated by red squares, Northern lineage; blue circles, Central lineage; and yellow triangle, Southern lineage. Population abbreviations are as in Table 1.

The *CR* data revealed three distinct lineages within *P. penicillata*, all with posterior support of 100% (Fig. 3), corresponding to populations in discrete geographic regions: northeastern NSW/southeast Queensland (=Northern lineage), central NSW (= Central lineage) and Victoria (=Southern lineage) (Fig. 2). The mean pairwise sequence divergence among the three major lineages was 7.1%, with only limited divergence within lineages (mean 1.6%: Table 3). There was no obvious geographic structure within the Southern or Central lineages, but within the Northern lineage, the sequences from southeast Queensland and far northeastern NSW tended to cluster to the exclusion of samples from further south (Amr, Wo1, Wo2), albeit with poor support (posterior probability of 0.59). The three major lineages in *P. penicillata* formed a polytomy along with the lineage that gave rise to *P. herberti*, with no support for reciprocal monophyly of the two species. When the haplotype analysis in MrBayes was rerun to include a topological constraint enforcing monophyly of *P. penicillata* haplotypes, the resulting model was slightly worse (Bayes factor test, 2ΔlnHML = −7.9), supporting the presence of a 4-way polytomy within the *P. penicillata*/*P. herberti* complex. Molecular clock dating suggests the major lineages within the *P. penicillata*/*P. herberti* complex initially diverged in the early Pleistocene (Fig. 3).

**Table 3:** Average sequence divergence ± SE, within (on diagonal) and between (below diagonal) the three major mtDNA control region lineages identified within brush-tailed rock-wallabies.

	Northern	Central	Southern
Northern	0.013 ± 0.0005		
Central	0.053 ± 0.0004	0.022 ± 0.0012	
Southern	0.072 ± 0.0007	0.088 ± 0.0009	0.013 ± 0.0018

Most pairwise Φ_*ST*_ estimates were significant and high, averaging 0.84 ± 0.02 (SE); range 0.00–1.00 (Table 4). Exceptions were for colonies in close proximity that shared haplotypes (Tables 1 and 3). Comparisons between populations among the three mtDNA lineages were high (mean ± SE: 0.95 ± 0.01). In contrast, the within lineage Φ_*ST*_ estimates were more moderate (0.64 ± 0.05). The AMOVA examining genetic variation at different hierarchical levels revealed that most of the total molecular variance was partitioned among the three identified phylogeographic regions (72%, Φct = 0.72, *P *<* *0.0001), with 23% partitioned among colonies within regions (Φsc = 0.82, *P *<* *0.0001). Only 5% of total molecular variance partitioned within colonies (Φst = 0.95, *P *<* *0.0001), as could be expected given the large number of sample sites represented by only a single haplotype.

**Table 4:** Pairwise Φ_*ST*_ estimates (below diagonal) among 14 sampled brush-tailed rock-wallaby colonies in southeastern Australia.

Site	CN-Q	PD-Q	EC-Q	FC-Q	FCE-Q	HC-Q	Arm-N	Mar-N	DC-N	IR-N	BR-N	JC-N	RPC-V	Mer-V
CN-Q		NS	*	*	NS	*	*	*	*	*	*	*	*	*
PD-Q	0.12		*	*	NS	*	*	*	*	*	*	*	*	*
EC-Q	0.63	0.79		*	*	NS	*	*	*	*	*	*	*	*
FC-Q	0.57	0.80	1.00		NS	*	*	*	*	*	*	*	*	*
FCE-Q	0.32	0.60	0.76	0.34		NS	*	NS	*	*	*	*	*	NS
HC-Q	0.37	0.56	0.20	0.33	0.14		*	*	*	*	*	*	*	*
Arm-N	0.76	0.80	0.91	0.92	0.82	0.76		*	*	*	*	*	*	*
Mar-N	0.82	0.84	0.85	0.89	0.79	0.85	0.87		*	*	*	*	*	*
DC-N	0.96	0.97	1.00	1.00	0.99	0.92	0.98	0.73		*	*	*	*	*
IR-N	0.96	0.98	1.00	1.00	0.99	0.92	0.98	0.55	1.00		NS	*	*	*
BR-N	0.95	0.97	1.00	1.00	0.98	0.91	0.98	0.47	1.00	0.00		*	*	*
JC-N	0.97	0.98	1.00	1.00	0.99	0.94	0.99	0.93	1.00	1.00	1.00		*	*
RPC-V	0.95	0.96	1.00	1.00	0.98	0.93	0.98	0.92	1.00	1.00	1.00	1.00		*
Mer-V	0.91	0.92	0.94	0.95	0.90	0.91	0.94	0.87	0.98	0.98	0.98	0.99	0.44	

* Significance (*P* < 0.5) denoted above the diagonal. Abbreviations of sites are as in Table 1.

For *P. penicillata CR* haplotypes, a significant pattern of isolation by distance was found between genetic and geographical distances (Mantel test, *r* = 0.492, *P *<* *0.0001). This significant isolation by distance spatial patterning was also present within each of the examined mtDNA lineages (Mantel tests: Central *r* = 0.211, *P *<* *0.05; Northern *r* = 0.578, *P *=* *001). Mismatch distributions for all haplotypes and for haplotypes within the Southern and Northern lineages were consistent with the demographic expansion model (*P *>* *0.05: Table 5). In contrast, the demographic expansion model was rejected for the Central lineage (*P *<* *0.05: Table 5). However, values of Fu's Fs or Tajima's D did not deviate significantly from neutrality in any population (Table 5).

**Table 5:** Mismatch distribution and neutrality tests for control region sequences from identified brush-tailed rock-wallaby mtDNA lineages.

	Mismatch distribution		Neutrality tests	
	SSD	R	Tajima's D	Fu's F
Northern	0.0230 (0.137)	0.0453 (0.072)	0.0730 (0.607)	0.415 (0.614)
Central	**0.127** (0.012)	**0.240** (0.000)	0.929 (0.851)	7.498 (0.955)
Southern	0.0923 (0.375)	0.223 (0.070)	−0.251 (0.447)	1.713 (0.822)
Overall	0.017 (0.087)	**0.032** (0.000)	1.101 (0.894)	6.699 (0.896)

*P* values are in parentheses; significant (*P* < 0.5) values are indicated in bold; SSD, sum of squared deviations; R, Harpending raggedness index.

### Population genetic structure inferred from microsatellites

A total of 246 individuals from 14 populations were genotyped at 11 microsatellite loci (Table 2). All pairwise locus combinations were in linkage disequilibrium (*n* = 704 comparisons, *α *= 0.05, *P *=* *0.002 − 1), except the Pa595 and Pa385 combination in Drews Creek and Bowmans Road (*P *=* *0.00). None of the loci deviated significantly from HWE in any of the 14 sampled colonies after table-wide sequential Bonferroni corrections. Loci were moderately polymorphic, with 6–18 alleles per locus (*AD*: mean ± SE: 13.4 ± 1.0; *AR*: 3.2 ± 0.07; Table 2). Unbiased *H*_*E*_ estimates were moderate for most sites (0.52–0.70); however, two locations showed lower levels (Jenolan Caves – 0.49; Rocky Plains Creek – 0.33; Table 2).

Most pairwise *F*_*ST*_ estimates were significant, following adjustment for multiple comparisons (Table 6). The exceptions included the comparison between the Hurdle Creek and Farm Creek colonies, which were only 1 km apart, and all comparisons with the Martindale colony, the site with the lowest sample size (*n* = 7; Table 2). Pairwise *F*_*ST*_ values varied from 0.013 to 0.490 (Table 6). Comparisons between populations among the three mtDNA lineages were very high (0.337 ± 0.010), with the most extreme between Northern and Southern lineage populations (0.380 ± 0.018). In comparison, the within lineage *F*_*ST*_ estimates were moderate (0.196 ± 0.015), with Northern lineage populations showing the lowest levels of differentiation (0.179 ± 0.020). A Mantel test detected a strong positive correlation between geographic distance and genetic differentiation among the 14 populations (*r *=* *0.732, *P* < 0.01), with ∼54% of the variation in genetic differentiation explained by distance among the sampled colonies (*r*^*2*^ = 0.536).

**Table 6:** Pairwise F_ST_ estimates (below diagonal) among 14 sampled brush-tailed rock-wallaby colonies in southeastern Australia.

Site	CN-Q	PD-Q	EC-Q	FC-Q	FCE-Q	HC-Q	Arm-N	Mar-N	DC-N	IR-N	BR-N	JC-N	RPC-V	Mer-V
CN-Q		*	*	*	*	*	*	NS	*	*	*	*	*	*
PD-Q	0.069		*	*	*	*	*	NS	*	*	*	*	*	*
EC-Q	0.185	0.182		*	*	*	*	NS	*	*	*	*	*	*
FC-Q	0.233	0.184	0.097		*	*	*	NS	*	*	*	*	*	*
FCE-Q	0.240	0.218	0.092	0.065		*	*	NS	*	*	*	*	*	*
HC-Q	0.186	0.172	0.074	0.013	0.065		*	NS	*	*	*	*	*	*
Arm-N	0.340	0.292	0.242	0.261	0.295	0.259		NS	*	*	*	*	*	*
Mar-N	0.331	0.290	0.224	0.240	0.268	0.240	0.225		NS	NS	NS	NS	NS	NS
DC-N	0.391	0.370	0.338	0.343	0.340	0.328	0.311	0.225		*	*	*	*	*
IR-N	0.324	0.294	0.275	0.258	0.291	0.245	0.256	0.184	0.191		*	*	*	*
BR-N	0.336	0.304	0.288	0.280	0.310	0.265	0.260	0.198	0.210	0.093		*	*	*
JC-N	0.478	0.446	0.421	0.414	0.441	0.375	0.385	0.304	0.310	0.255	0.303		*	*
RPC-V	0.490	0.462	0.404	0.437	0.457	0.358	0.435	0.408	0.438	0.294	0.323	0.477		*
Mer-V	0.383	0.358	0.283	0.311	0.345	0.281	0.313	0.233	0.338	0.225	0.250	0.376	0.245	

* Significance (*P* < 0.05) denoted above the diagonal. Abbreviations of sites are as in Table 1.

**Figure 4:**
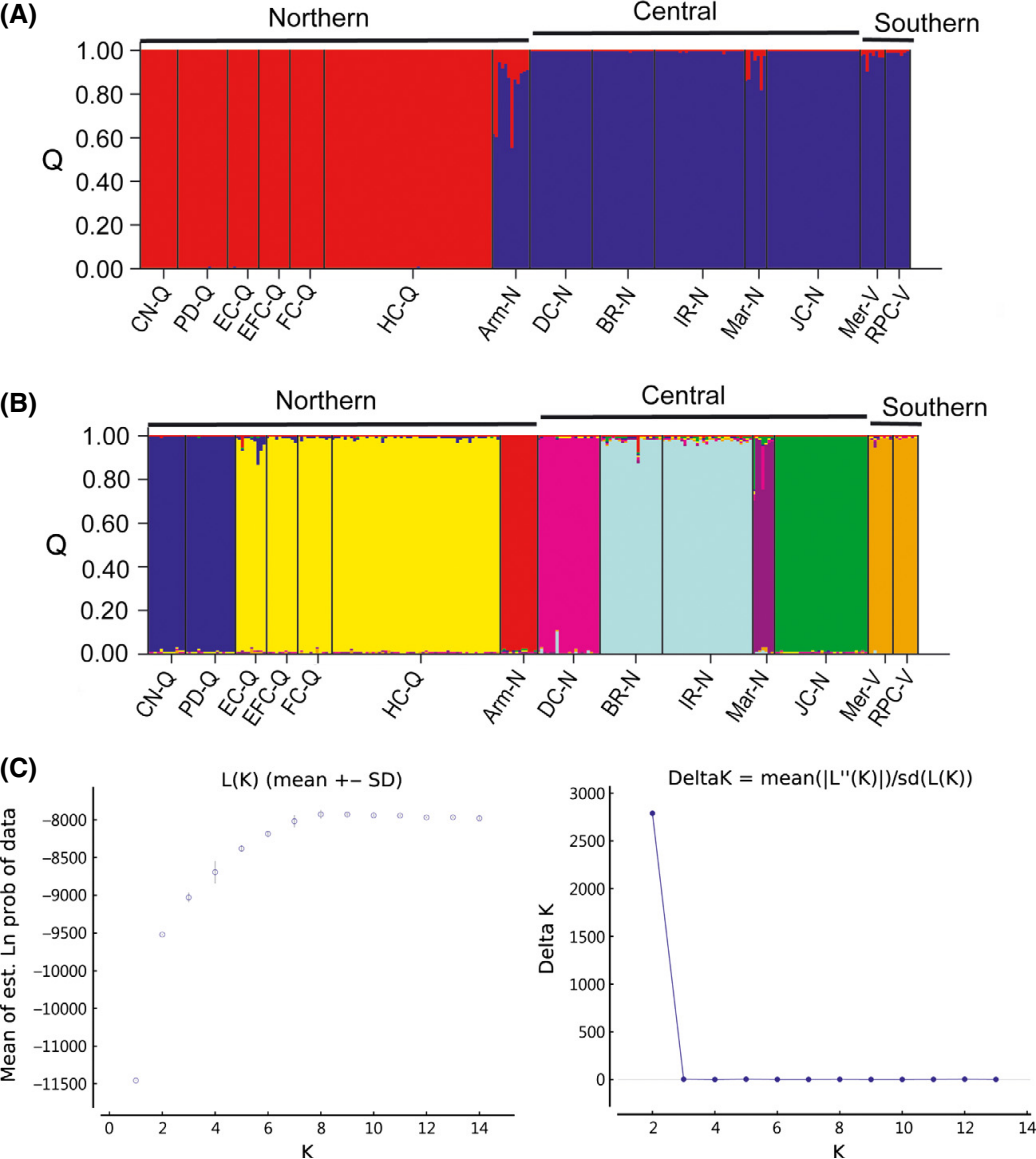
Stucture plots showing proportions of inferred ancestry (Q) in the genetic clusters identified within brush-tailed rock-wallabies sampled from 14 sites, when (A) K = 2 or (B) K = 8. (C) Structure output showing maximum *L*(*K*) at K = 8 and maximum Δ*K* at K = 2. See Table 1 for population abbreviations. (A) Identified clusters when K = 2 (B) Identified clusters when K = 8. (C) Graphs of Structure output showing maximum *L*(*K*) at K = 8 and maximum Δ*K* at K = 2.

**Figure 5:**
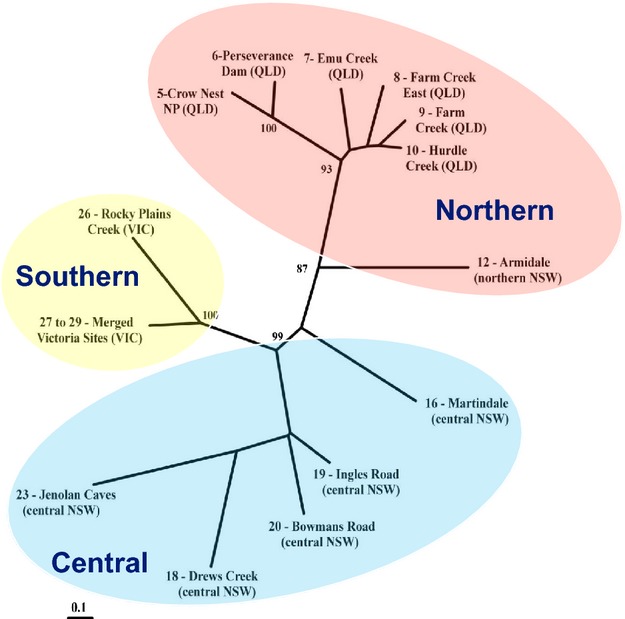
Unrooted neighbor-joining tree based on average microsatellite allele sharing genetic distances (Dps) among sampled brush-tailed rock-wallaby colonies in southeastern Australia. See Fig. 2 for population locations and Table 1 for population identity numbers. The clustering of populations with respect to the three identified mtDNA lineages is indicated by shading: red, Northern lineage; blue, Central lineage; and yellow, Southern lineage.

The Bayesian model-based clustering analysis implemented in STRUCTURE indicated that either eight (maximum *L*(*K*)) or two (maximum Δ*K*) populations were present in the complete data set (Fig. 4). With K = 2, these inferred populations corresponded to all 6 geographic populations sampled from Queensland (sites 5–10; Table 2) in one cluster and the remaining eight geographic populations sampled from New South Wales and Victoria (sites 12–29; Table 2) in a second (Fig. 3A). With K = 8, the samples from Qld were separated into two clusters (sites 5, 6 in a northern and sites 7–10 in a southern cluster), while the NSW/Vic populations were separated into six regional clusters corresponding to sites 12, 16, 18, 19–20, 23, 26–29 (Fig. 3B). When the K = 2 Qld and NSW/Vic clusters were analyzed independently, the substructuring identified the same clusters detected with K = 8, except for the two Watagans populations (sites 19, 20), which showed increased separation in the later analysis (Supplementary Figs S1 and S2). Finally, the unrooted neighbor-joining tree, based on average allele sharing (Dps) among the 14 sampled colonies, depicted three distinct clusters, which correspond to the Northern, Central and Southern lineages (Fig. 5).

## Discussion

Our mtDNA and microsatellite analysis found brush-tailed rock-wallaby populations to be highly structured both locally and regionally throughout southeastern Australia. Our data supported the three geographically discreet and divergent *CR* lineages. These major regional groupings were also supported by *F*_*ST*_ and clustering analyses of the microsatellite data, and provide evidence for long-term barriers to gene flow. While the deep phylogenetic break between the Northern and Central lineages was found to coincide with a known biogeographic barrier (i.e., the Hunter Valley), the major break between the Central and Southern lineages did not coincide with any known barrier.

### Hunter Valley barrier

The broad (30–50 km) and comparatively dry lowlands of the Hunter River Valley in central NSW create a major break in the Great Dividing Range from the coast ∼180 km west to the western slopes (Short et al. 1983) and form a well-established biogeographic barrier in southeastern Australia (Cracraft 1991; Schodde and Mason 1999). Even today, it provides the only access to the east coast for inland taxa and a northerly, or southerly, limit to the distribution of multiple mesic (sensu Byrne et al. 2011) species (Short et al. 1983). Its impact in cooler and drier glacial cycles will therefore have been even more significant, as is evidenced by major phylogenetic breaks across this barrier not just in brush-tailed rock-wallabies, but in a variety of now more continuously distributed mesic vertebrate species including birds (Joseph et al. 2008), reptiles (Chapple et al. 2005, 2011a,2011b; Moussalli et al. 2005; Dubey and Shine 2010), and amphibians (Donnellan et al. 1999; Schauble and Moritz 2001). The divergence in brush-tailed rock-wallabies suggests that some mid-Pleistocene arid cycles were sufficiently severe to even impact species such as the brush-tailed rock-wallaby that can tolerate much drier conditions (dry schlerophyll forest and woodland) than most species studied to date. This suggests that at least two major mesic refugia were present in eastern Australia during Pleistocene glacial cycles. Phylogeographic analysis of another woodland marsupial species (*Petaurus breviceps*) also found evidence for an eastern coastal Plio-Pleistocene refugium (Malekian et al. 2010).

### Southern NSW barrier = Great Dividing Range barrier?

The second major phylogeographic break indentified in brush-tailed rock-wallabies occurs in southern NSW, between Sydney and the NSW/Vic border. Its precise location is unclear because populations of brush-tailed rock-wallabies became extinct in the region south of Taralga (NSW) and north of East Gippsland (Vic) late last century (Dovey et al. 1997; Lunney et al. 1997). However, Paplinska et al. (2011) used museum specimens to extend the known range of the Central lineage to south of the ACT (Fig 1). While no obvious north–south barrier to gene flow occurs in this region, it is emerging as the location of major phylogenetic breaks within a variety of vertebrate species including lizards (Chapple et al. 2005, 2011a), mammals (Frankham et al. 2012), frogs (Schauble and Moritz 2001; Symula et al. 2008), and as a contact zone between sibling species (e.g., Dickman et al. 1988; Donnellan et al. 1999; Lindenmayer et al. 2002; Burns and Crayn 2006). This geographically coincident phylogenetic break in a variety of taxa strongly suggests a common underlying cause. The periodic flooding of the Gippsland Basin has been suggested as a mechanism (Chapple et al. 2005), although this region may be too far south to be a valid explanation for some of these taxa, and in addition is unlikely to have impacted species such as brush-tailed rock-wallabies that are mainly confined to the ranges and have no record (historic or subfossil) of occurrence south of the Gippsland Basin (Menkhorst 1995). In addition, the last major inundation occurred too early (late Miocene – early Pliocene) to impact brush-tailed rock-wallabies (Chapple et al. 2005).

The NSW/Vic border region does, however, contain the highest mountains in Australia (the Australian Alps up to 2228 m), which were the site of most glaciation that occurred in Australia during Pleistocene arid cycles (Barrows et al. 2001). Unlike in the Northern Hemisphere, the actual area of the Australian Alps under glacial ice was small (∼15 km^2^), nevertheless the periglacial zone of largely treeless steppe was much larger, extending in some glacial cycles down to 1000 m (reviewed in Garrick et al. 2004), rendering much of the Great Dividing Range in this region uninhabitable for mesic forest taxa. When combined with the narrowness of the immediately adjacent eastern coastal slopes and plain (Fig. 2), we hypothesize that a significant north–south barrier to gene flow was created for a variety of mesic taxa, mostly of low vagility. These taxa nevertheless, persisted in less hostile areas to the north and south. The Great Dividing Range (from the Blue Mountains to the Australian Alps) has previously been identified as a significant current and historic barrier (GDR barrier Ford 1986) to east–west gene flow in a variety of vertebrates (Ford 1986; Chapple et al. 2005, 2011a,2011b; Nicholls and Austin 2005; Symula et al. 2008).

Our data therefore support the existence of three major refugia for brush-tailed rock-wallabies in southeastern Australia during the Pleistocene – one north and one south of the Hunter River Valley, and a third south of the Australian Alps. While there are concordant breaks in some codistributed taxa, additional studies of other widespread southeastern Australian taxa from a variety of habitats are now required to test the generality of this hypothesis. Interestingly, divergences across the Hunter and Southern Australian Barriers in some codistributed taxa are considerably older and deeper (Pliocene–Miocene) than those reported here for brush-tailed rock-wallabies (mid-Pliestocene), reinforcing the long-term impacts of these barriers on the fauna of southeastern Australia (Chapple et al. 2005, 2011a,2011b; Symula et al. 2008; Frankham et al. 2012).

### Intralineage divergence and structure

Although the *CR* divergence among the three brush-tailed rock-wallaby lineages was high (∼7%), intralineage divergence was modest (∼1.6%) and mostly lacked geographic structuring. While this pattern is consistent with expansion and recolonization from refugia, molecular evidence for a recent expansion is inconclusive (Table 5). As it is likely that Pleistocene aridity cycles differed in their intensity, the impact of these barriers may have accumulated over successive cycles (Byrne et al. 2011) and any major expansion may not be recent. Evidence for additional geographic substructuring is only present within the Northern lineage with samples from northern and southern areas clustering separately for both mtDNA and microsatellite data. This suggests that in this region multiple small-scale refugia existed during less severe glacial cycles. Similar patterns of substructuring north and south of the Clarence River Valley are seen in the codistributed long-nosed potoroo (*Potorous tridactylus*) (Frankham et al. 2012) and Hastings River mouse (*Pseudomys oralis*) (Rowe et al. 2011), providing evidence for the existence of “refugia within refugia” in this region.

Within each lineage, brush-tailed rock-wallabies show high levels of local divergence for both mtDNA and microsatellite data. *CR* haplotypes were rarely shared between populations except those in close proximity (∼4 km), and high Φ_*ST*_ values were found among almost all populations. These data support the high levels of female philopatry previously reported in this species (Hazlitt et al. 2004, 2010). Populations were also highly structured for microsatellites, even when in close proximity (∼4 km), with high *F*_*ST*_ values typical and most populations being identified as distinct clusters in the STRUCTURE analysis (Fig 3). However, as most of these populations retain levels of genetic diversity typical for *Petrogale,* we can assume that they were connected by low level, male-mediated gene flow at least historically (Eldridge et al. 2010). In contrast, isolated remnant populations like those in Vic and at Jenolan Caves have lower diversity (Table 2), most likely as a consequence of genetic drift and isolation.

### Interspecific relationships

Our *CR* data were unable to resolve relationships among the three brush-tailed rock-wallaby lineages and *P. herberti*, the sister species to *P. penicillata* (Potter et al. 2012a). This lack of distinctiveness is also reflected in the introgression of allozyme alleles and mtDNA RFLPs across their hybrid zone in southeast Qld (Eldridge and Close 1992; Bee and Close 1993). So, although *P. penicillata* and *P. herberti* have distinct karyotypes (Eldridge et al. 1990), the mtDNA data indicate that the three major lineages within *P. penicillata* are as distinct from each other as any are from *P. herberti*. Divergence among these four lineages appears to date from the early-mid-Pleistocene, a conclusion previously reached for the divergence between *P. penicillata* and *P. herberti* and other eastern *Petrogale* species (Campeau-Peloquin et al. 2001; Potter et al. 2012a). Why some divergences have resulted in speciation (and associated chromosome evolution) and others only in deep intraspecific structure is intriguing but remains uncertain.

### Implications for management

The three distinct genetic lineages identified within brush-tailed rock-wallabies in this study appear to reflect Pleistocene divergences and are not the result of a recent anthropogenically induced decline. These lineages show reciprocal monophyly for their mtDNA and significant divergence at microsatellite loci and thus would each equate to an evolutionarily significant unit (ESU) (sensu Moritz 1994). As each ESU contains unique diversity, we would suggest that brush-tailed rock-wallabies be managed to maximize the retention of genetic variation. This should not exclude mixing individuals from different ESUs, for example to supplement the “critically endangered” southern ESU, which is now restricted to a single wild population (*n* < 30) in eastern Victoria (Bluff et al. 2011). However, given the apparent long-term separation of the ESUs and the broad latitudinal range of the species >1000 km, local adaptation and therefore outbreeding depression cannot be excluded (Frankham et al. 2011). Therefore, any interlineage mixing should be monitored.

## Supporting Information

**Figure S1.** Stucture plots showing proportion of inferred ancestry (Q) in the genetic clusters identified within the Qld brush-tailed rock-wallabies sampled from six sites. **Figure S2.** Structure plots showing proportion of inferred ancestry (Q) in the genetic clusters identified within the NSW/Vic brush-tailed rock-wallabies sampled from eight sites. See Table 2 for population codes and Fig. 1 for location of sites.
