# Supplementary material for: Three divergent lineages within an Australian marsupial (Petrogale penicillata) suggest multiple major refugia for mesic taxa in southeast Australia
Source: Ecol Evol. 2014 Mar 6;4(7):1102–16. doi: 10.1002/ece3.1009 (PMC3997325; doi:10.1002/ece3.1009)
Supplement: Supplementary file 1 — Figure S1. Stucture plots showing proportion of inferred ancestry (Q) in the genetic clusters identified within the Qld brush-tailed rock-wallabies sampled from six sites. Figure S2. Structure plots showing proportion of inferred ancestry (Q) in the genetic clusters identified within the NSW/Vic brush-tailed rock-wallabies sampled from eight sites. See Table 2 for population codes and Fig. 1 for location of sites. [file ece30004-1102-sd1.doc]

Supplementary

**Supplementary Figure S1**. Stucture plots showing proportion of inferred ancestry (Q) in the genetic clusters identified within the Qld brush-tailed rock-wallabies sampled from 6 sites. See Table 2 for population codes and Figure 1 for location of sites.

(A) Graphs of Structure output showing maximum *L(K)* at K=2 and maximum Δ*K* at K=3.


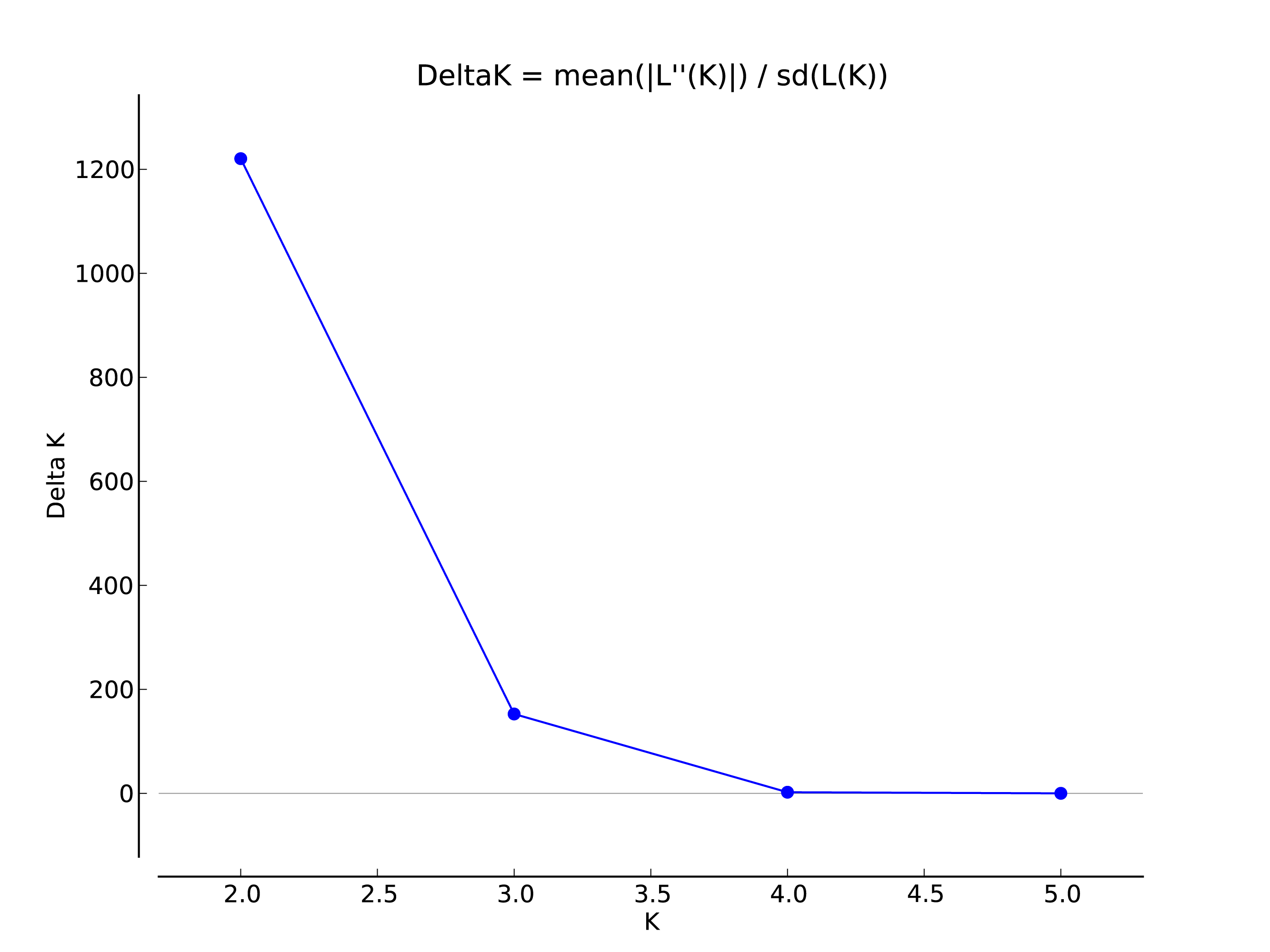

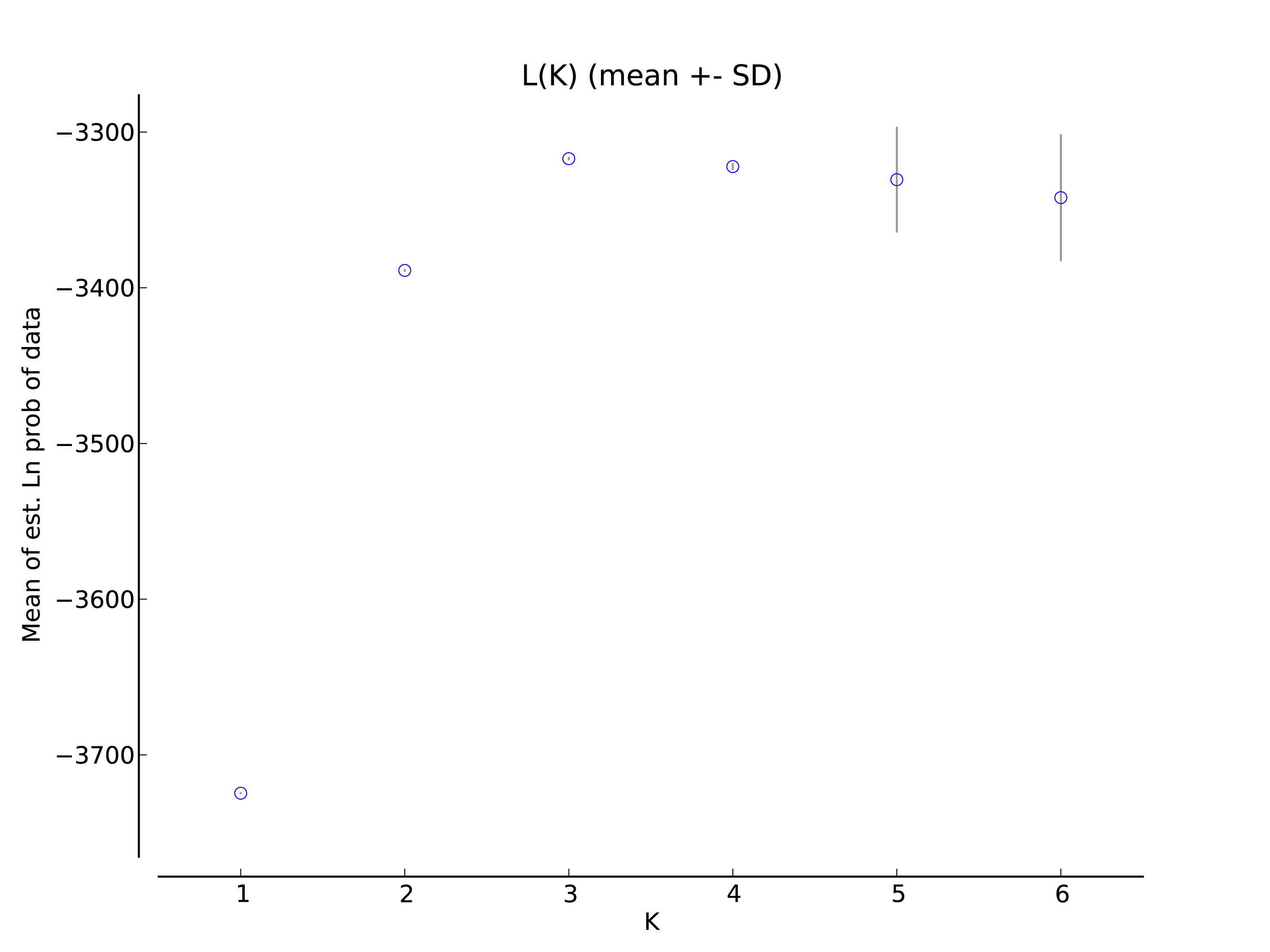


(B) Identified cluster when K=2


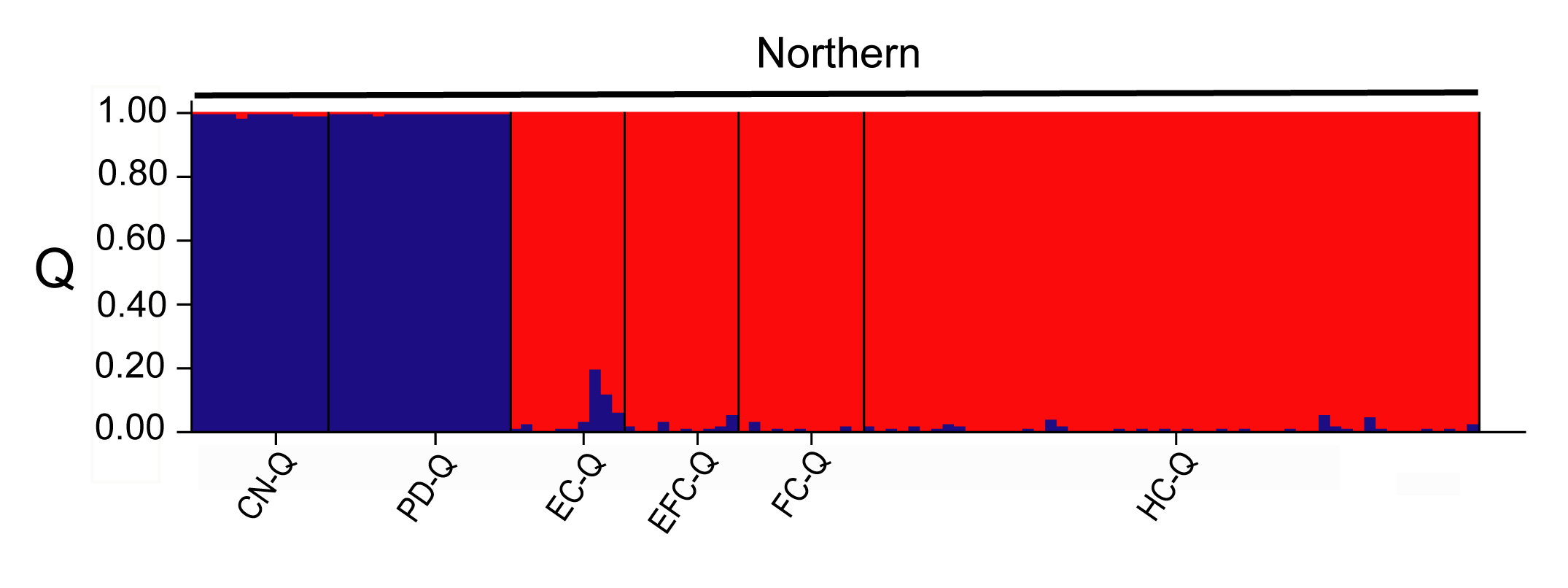


c) Identified clusters when K=3


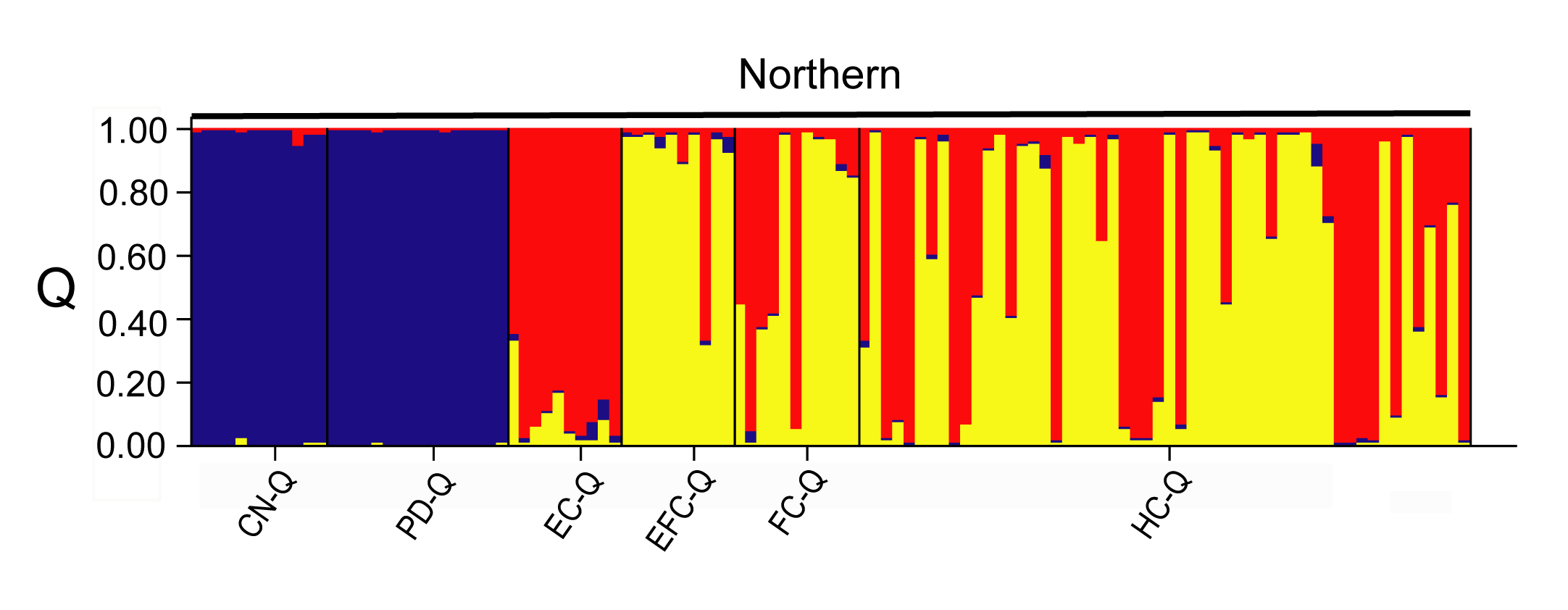


**Supplementary Figure S2**. Stucture plots showing proportion of inferred ancestry (Q) in the genetic clusters identified within the NSW/Vic brush-tailed rock-wallabies sampled from 8 sites. See Table 2 for population codes and Figure 1 for location of sites.

(A) Graphs of Structure output showing maximum *L(K)* and Δ*K* at K=7.


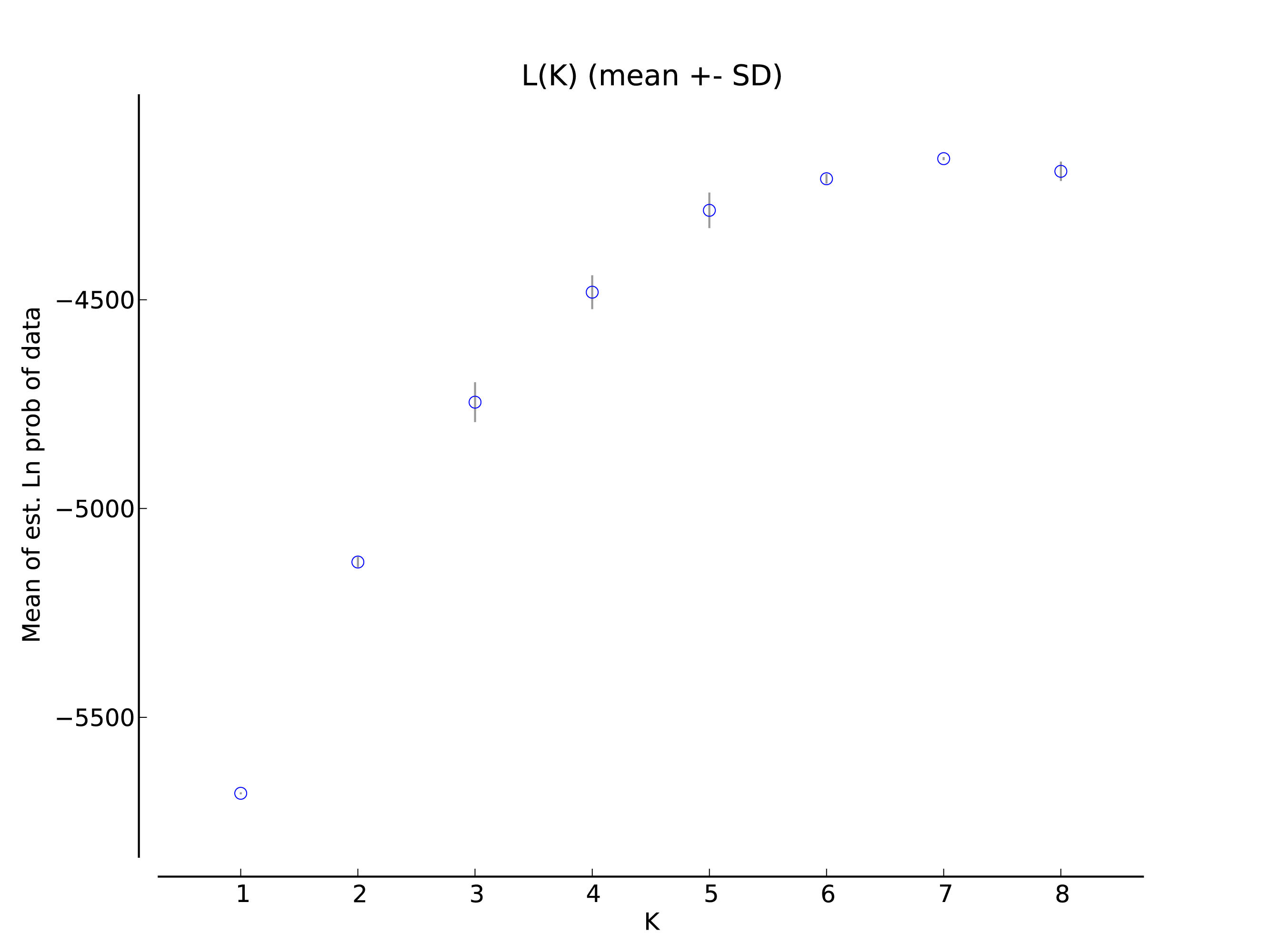

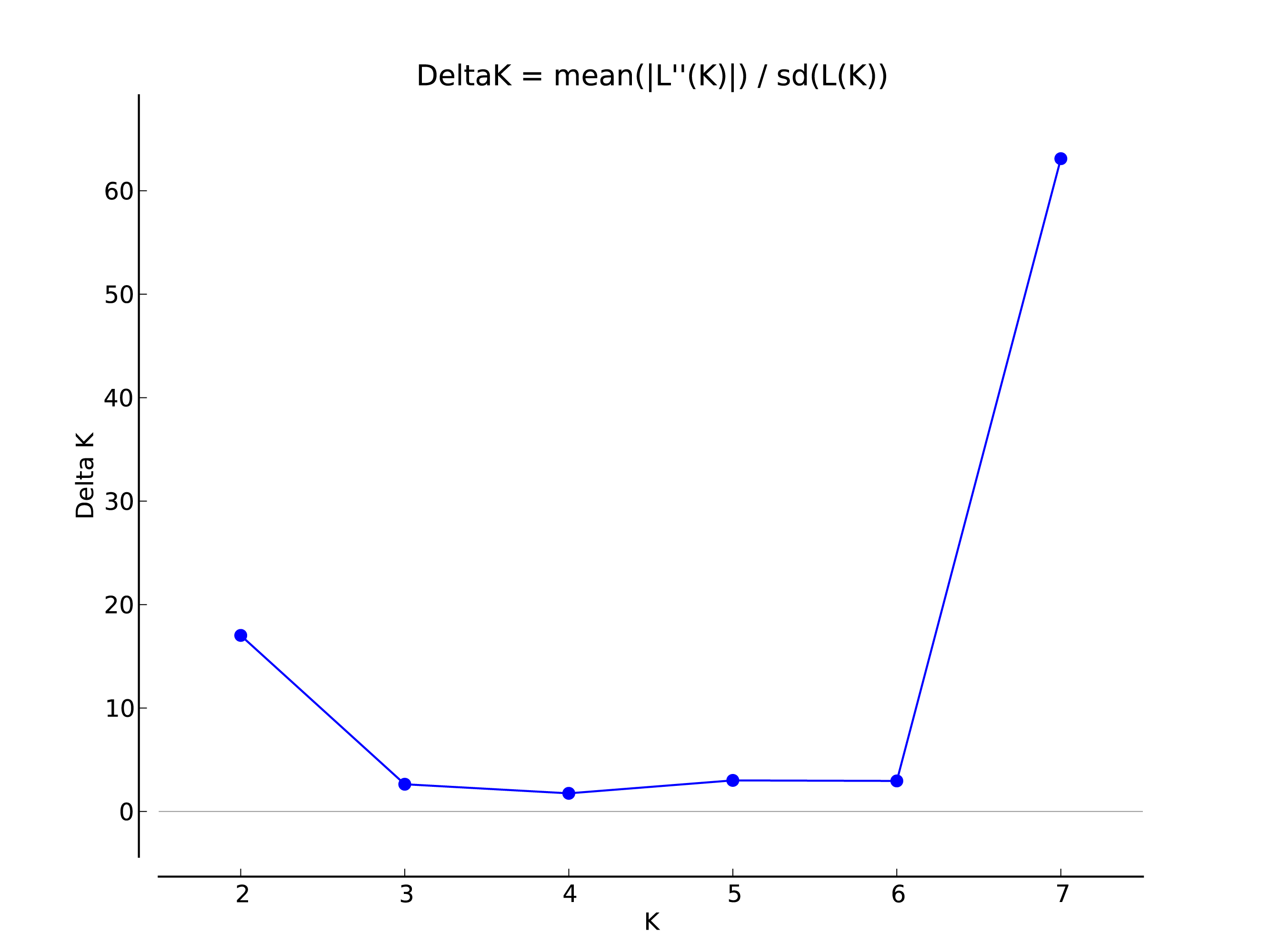


b) K=7

(B) Identified clusters when K=7


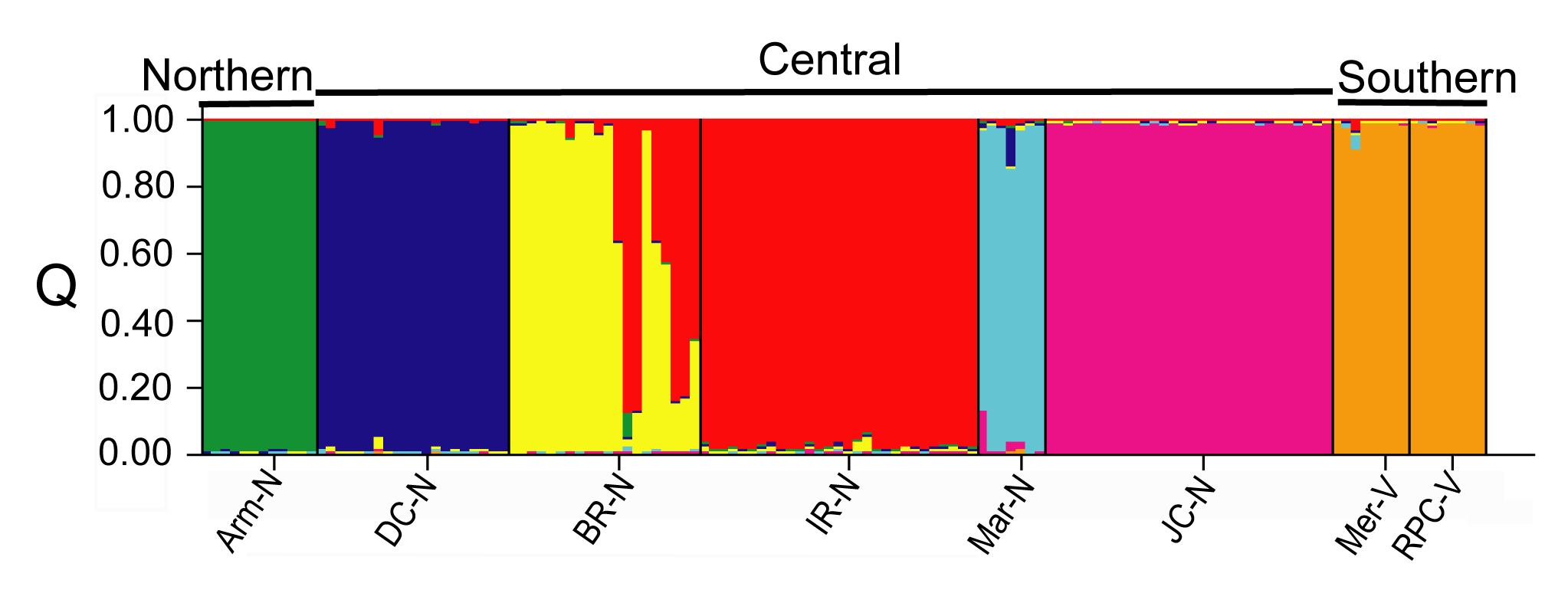


**Supplementary Table 1**. Observed allele frequencies, at 11 polymorphic microsatellite loci, in sampled *P. penicillata* populations. See Table 2 for population codes and Figure 1 for location of sites.

| **Locus** | **Allele (bp)** | **CN-Q** | **PD-Q** | **EC-Q** | **FC-Q** | **FCE-Q** | **HC-Q** | **Arm-N** | **Mar-N** | **DC-N** | **IR-N** | **BR-N** | **Jen-N** | **RPC-V** | **Mer-V** |
| --- | --- | --- | --- | --- | --- | --- | --- | --- | --- | --- | --- | --- | --- | --- | --- |
| Pa55 | 139 |  |  | 0.10 |  | 0.35 | 0.12 |  | 0.50 | 0.20 | 0.14 | 0.05 |  |  |  |
|  | 149 |  |  |  |  |  | 0.04 |  | 0.21 |  | 0.33 | 0.70 |  |  |  |
|  | 151 |  |  | 0.20 | 0.41 | 0.30 | 0.47 | 0.21 |  |  | 0.52 | 0.25 | 0.82 | 0.69 | 0.88 |
|  | 153 | 0.79 | 0.72 |  | 0.09 | 0.20 | 0.03 | 0.17 |  |  |  |  |  |  |  |
|  | 155 |  |  |  |  |  |  | 0.33 |  |  |  |  |  |  |  |
|  | 157 |  |  |  |  |  |  |  |  | 0.43 | 0.02 |  | 0.18 |  |  |
|  | 159 |  |  |  |  |  |  |  |  | 0.05 |  |  |  |  |  |
|  | 161 |  |  |  |  |  |  |  | 0.07 |  |  |  |  |  | 0.06 |
|  | 163 |  |  |  |  |  |  | 0.29 | 0.21 | 0.33 |  |  |  | 0.31 | 0.06 |
|  | 167 |  |  |  |  |  | 0.03 |  |  |  |  |  |  |  |  |
|  | 169 | 0.21 | 0.28 | 0.50 | 0.05 | 0.10 | 0.08 |  |  |  |  |  |  |  |  |
|  | 171 |  |  | 0.20 | 0.45 | 0.05 | 0.23 |  |  |  |  |  |  |  |  |
| Pa297 | 102 | 0.63 | 0.33 | 0.50 | 0.09 | 0.25 | 0.27 |  |  |  |  |  |  |  |  |
|  | 112 |  |  |  |  |  |  |  |  |  | 0.10 |  |  |  |  |
|  | 118 | 0.08 |  | 0.10 |  |  | 0.04 |  |  |  | 0.19 | 0.03 |  |  |  |
|  | 120 |  |  |  |  |  |  | 0.33 |  |  |  | 0.08 |  |  |  |
|  | 122 |  |  |  | 0.14 | 0.10 | 0.04 |  |  | 0.18 |  |  |  |  | 0.13 |
|  | 124 | 0.29 | 0.67 | 0.30 | 0.77 | 0.65 | 0.63 |  | 0.21 |  |  |  |  |  |  |
|  | 126 |  |  | 0.10 |  |  | 0.01 | 0.46 | 0.43 |  |  |  | 0.48 |  |  |
|  | 128 |  |  |  |  |  |  |  | 0.07 |  | 0.19 | 0.20 |  |  | 0.13 |
|  | 130 |  |  |  |  |  |  | 0.21 | 0.29 | 0.40 | 0.34 | 0.45 | 0.52 | 1.00 | 0.75 |
|  | 132 |  |  |  |  |  |  |  |  |  | 0.02 |  |  |  |  |
|  | 134 |  |  |  |  |  | 0.02 |  |  | 0.30 | 0.16 | 0.25 |  |  |  |
|  | 136 |  |  |  |  |  |  |  |  | 0.13 |  |  |  |  |  |
| Pa385 | 147 | 0.54 | 0.50 | 0.85 | 0.73 | 1.00 | 0.63 |  |  |  |  |  |  |  |  |
|  | 149 | 0.04 |  | 0.15 | 0.18 |  | 0.25 |  |  |  |  |  |  |  |  |
|  | 151 |  |  |  |  |  | 0.01 |  | 0.64 |  |  | 0.08 |  |  |  |
|  | 153 |  |  |  | 0.05 |  | 0.10 | 0.25 |  |  | 0.10 | 0.18 | 0.38 |  |  |
|  | 155 |  |  |  |  |  |  | 0.46 | 0.14 | 0.15 | 0.17 | 0.28 | 0.35 | 0.69 | 0.63 |
|  | 157 |  |  |  |  |  |  | 0.29 |  | 0.13 | 0.53 | 0.18 |  | 0.31 | 0.38 |
|  | 159 |  |  |  | 0.05 |  | 0.01 |  |  |  |  | 0.08 |  |  |  |
|  | 161 | 0.42 | 0.09 |  |  |  |  |  |  | 0.28 |  |  |  |  |  |
|  | 163 |  |  |  |  |  |  |  | 0.21 | 0.23 |  | 0.10 | 0.27 |  |  |
|  | 165 |  | 0.38 |  |  |  |  |  |  |  | 0.19 | 0.05 |  |  |  |
|  | 167 |  | 0.03 |  |  |  |  |  |  | 0.23 |  |  |  |  |  |
|  | 169 |  |  |  |  |  |  |  |  |  |  | 0.08 |  |  |  |
| Pa593 | 119 |  |  |  |  |  |  | 0.21 |  |  |  |  |  |  |  |
|  | 125 | 0.42 | 0.22 | 0.05 |  |  |  |  |  |  |  |  |  |  |  |
|  | 127 | 0.04 |  |  |  |  |  | 0.25 |  |  | 0.07 |  |  |  |  |
|  | 129 |  |  |  |  |  |  |  | 0.07 | 0.05 | 0.23 | 0.30 |  | 0.31 | 0.19 |
|  | 131 |  |  | 0.05 | 0.32 | 0.30 | 0.48 |  |  |  | 0.20 | 0.23 |  |  | 0.06 |
|  | 133 |  |  |  |  |  |  |  | 0.36 |  |  | 0.10 | 0.42 |  |  |
|  | 135 | 0.08 | 0.19 |  | 0.05 |  | 0.02 | 0.08 | 0.21 | 0.34 | 0.45 | 0.28 | 0.58 |  |  |
|  | 137 |  |  |  |  |  | 0.02 |  |  | 0.53 |  | 0.08 |  |  |  |
|  | 139 |  |  | 0.45 | 0.27 | 0.35 | 0.25 | 0.33 | 0.14 |  |  |  |  |  |  |
|  | 141 | 0.08 | 0.03 | 0.25 | 0.09 | 0.05 | 0.10 |  | 0.21 | 0.08 |  |  |  |  | 0.19 |
|  | 143 | 0.38 | 0.41 | 0.10 |  |  | 0.09 | 0.13 |  |  |  |  |  |  | 0.31 |
|  | 145 |  | 0.16 | 0.05 |  |  |  |  |  |  |  |  |  |  |  |
|  | 147 |  |  | 0.05 | 0.27 | 0.30 | 0.04 |  |  |  |  |  |  | 0.69 |  |
|  | 151 |  |  |  |  |  |  |  |  |  | 0.05 | 0.03 |  |  | 0.06 |
|  | 153 |  |  |  |  |  |  |  |  |  |  |  |  |  | 0.19 |
| Pa595 | 203 |  |  |  |  |  |  |  |  | 0.08 |  | 0.18 |  |  |  |
|  | 207 |  |  | 0.05 |  |  |  |  | 0.29 | 0.18 |  |  | 0.27 |  | 0.44 |
|  | 211 |  |  |  |  |  | 0.02 | 0.04 | 0.36 | 0.21 | 0.09 |  |  |  |  |
|  | 215 | 0.14 | 0.16 |  | 0.14 | 0.05 | 0.23 | 0.08 | 0.21 | 0.24 | 0.25 |  |  |  |  |
|  | 219 | 0.14 |  | 0.25 | 0.05 | 0.25 | 0.21 |  |  |  | 0.16 | 0.25 | 0.02 | 1.00 | 0.06 |
|  | 223 | 0.05 |  | 0.30 | 0.45 | 0.55 | 0.26 | 0.21 |  |  | 0.04 |  |  |  | 0.19 |
|  | 227 |  | 0.16 |  | 0.09 | 0.05 | 0.04 | 0.17 | 0.07 |  | 0.14 | 0.40 | 0.62 |  |  |
|  | 231 | 0.23 | 0.19 | 0.15 | 0.14 | 0.05 | 0.11 | 0.21 | 0.07 | 0.05 | 0.27 | 0.13 |  |  | 0.31 |
|  | 235 | 0.09 | 0.25 | 0.10 | 0.14 | 0.05 | 0.02 | 0.29 |  |  | 0.05 |  |  |  |  |
|  | 239 | 0.23 | 0.25 |  |  |  | 0.03 |  |  |  |  |  | 0.10 |  |  |
|  | 243 | 0.14 |  | 0.15 |  |  | 0.08 |  |  |  |  | 0.05 |  |  |  |
|  | 251 |  |  |  |  |  |  |  |  | 0.24 |  |  |  |  |  |
| Pa597 | 92 | 0.29 | 0.28 | 0.35 | 0.09 | 0.40 | 0.12 |  |  |  |  |  |  |  |  |
|  | 96 |  |  |  |  |  |  |  |  |  |  |  |  |  | 0.31 |
|  | 100 | 0.38 | 0.34 | 0.50 | 0.64 | 0.60 | 0.69 | 0.08 |  |  | 0.04 | 0.08 |  |  |  |
|  | 102 | 0.04 | 0.06 |  |  |  |  |  | 0.43 | 0.65 | 0.21 | 0.18 | 0.35 | 0.06 |  |
|  | 104 | 0.29 | 0.31 | 0.15 | 0.18 |  | 0.13 | 0.17 |  |  |  |  |  |  |  |
|  | 106 |  |  |  |  |  |  | 0.38 |  | 0.25 | 0.05 | 0.33 |  |  |  |
|  | 110 |  |  |  | 0.09 |  | 0.06 |  |  |  |  |  |  |  |  |
|  | 112 |  |  |  |  |  |  |  |  |  | 0.07 | 0.30 | 0.03 |  |  |
|  | 116 |  |  |  |  |  |  |  |  | 0.10 |  |  | 0.62 |  |  |
|  | 118 |  |  |  |  |  |  |  | 0.07 |  |  | 0.03 |  |  |  |
|  | 120 |  |  |  |  |  |  |  |  |  |  |  |  |  | 0.06 |
|  | 122 |  |  |  |  |  |  |  |  |  |  |  |  |  | 0.25 |
|  | 126 |  |  |  |  |  |  |  |  |  |  |  |  | 0.56 | 0.25 |
|  | 128 |  |  |  |  |  |  |  |  |  |  |  |  | 0.38 | 0.13 |
|  | 132 |  |  |  |  |  |  |  | 0.36 |  | 0.34 | 0.10 |  |  |  |
|  | 136 |  |  |  |  |  |  |  | 0.14 |  | 0.29 |  |  |  |  |
|  | 140 |  |  |  |  |  |  | 0.13 |  |  |  |  |  |  |  |
|  | 142 |  |  |  |  |  |  | 0.25 |  |  |  |  |  |  |  |
| Me2 | 214 |  |  |  |  |  |  |  |  | 0.53 | 0.31 | 0.03 | 0.25 | 0.13 | 0.19 |
|  | 218 | 0.42 | 0.34 | 0.65 | 0.55 | 0.65 | 0.53 | 0.88 | 0.21 | 0.38 | 0.09 | 0.03 | 0.03 |  |  |
|  | 220 | 0.58 | 0.66 | 0.35 | 0.45 | 0.35 | 0.47 | 0.13 | 0.36 | 0.10 | 0.60 | 0.73 | 0.10 | 0.88 | 0.81 |
|  | 222 |  |  |  |  |  |  |  |  |  |  | 0.23 | 0.35 |  |  |
|  | 224 |  |  |  |  |  |  |  | 0.36 |  |  |  | 0.27 |  |  |
|  | 226 |  |  |  |  |  |  |  | 0.07 |  |  |  |  |  |  |
| Me14 | 162 |  |  |  |  |  |  |  |  |  | 0.02 | 0.13 |  |  |  |
|  | 164 |  |  |  |  |  |  |  |  |  |  | 0.08 |  |  |  |
|  | 168 |  |  |  |  |  | 0.01 | 0.08 |  |  |  |  | 0.26 |  |  |
|  | 174 |  | 0.09 |  |  |  |  |  |  | 0.05 |  |  |  |  |  |
|  | 176 | 1.00 | 0.84 | 0.40 | 0.40 | 0.15 | 0.44 |  |  |  | 0.07 | 0.13 |  | 0.25 | 0.06 |
|  | 178 |  |  | 0.45 | 0.05 |  | 0.04 | 0.58 | 0.50 | 0.03 |  |  |  |  |  |
|  | 180 |  |  |  | 0.05 | 0.05 | 0.11 |  |  | 0.11 | 0.41 | 0.20 |  | 0.75 |  |
|  | 182 |  | 0.06 |  | 0.15 |  | 0.13 |  |  | 0.34 | 0.36 |  | 0.45 |  |  |
|  | 184 |  |  |  | 0.10 | 0.15 | 0.06 |  | 0.50 | 0.39 |  |  |  |  | 0.50 |
|  | 186 |  |  |  | 0.10 | 0.15 | 0.13 |  |  | 0.03 | 0.14 | 0.20 | 0.16 |  |  |
|  | 188 |  |  | 0.10 | 0.15 | 0.50 | 0.09 |  |  | 0.03 |  |  | 0.09 |  | 0.19 |
|  | 190 |  |  |  |  |  |  | 0.33 |  | 0.03 |  | 0.28 | 0.05 |  | 0.25 |
|  | 196 |  |  | 0.05 |  |  |  |  |  |  |  |  |  |  |  |
| Me15 | 215 |  |  |  |  |  |  |  |  | 0.88 | 0.11 | 0.50 |  |  |  |
|  | 233 |  |  |  |  |  |  | 0.29 |  |  | 0.02 | 0.03 |  |  |  |
|  | 235 |  |  |  |  |  |  |  | 0.07 |  |  |  | 0.33 |  |  |
|  | 237 |  |  |  |  |  |  |  | 0.29 |  |  |  | 0.40 |  | 0.25 |
|  | 239 |  |  |  |  |  |  |  | 0.36 | 0.13 | 0.36 | 0.30 | 0.23 | 0.44 | 0.06 |
|  | 241 |  |  | 0.05 | 0.18 |  | 0.15 | 0.17 |  |  | 0.32 | 0.18 |  |  |  |
|  | 243 |  |  | 0.10 | 0.05 |  | 0.06 |  |  |  |  |  |  |  |  |
|  | 245 | 0.04 |  |  |  |  |  |  |  |  | 0.18 |  |  |  |  |
|  | 247 | 0.33 | 0.06 |  | 0.18 | 0.20 | 0.19 | 0.33 |  |  | 0.02 |  |  |  |  |
|  | 249 |  |  |  |  |  |  | 0.04 |  |  |  |  |  |  |  |
|  | 251 |  |  | 0.20 |  |  | 0.01 |  |  |  |  |  |  |  | 0.19 |
|  | 253 | 0.25 | 0.25 | 0.25 |  | 0.15 | 0.04 |  |  |  |  |  | 0.03 | 0.56 | 0.50 |
|  | 255 | 0.04 | 0.06 | 0.25 | 0.23 | 0.25 | 0.19 |  |  |  |  |  |  |  |  |
|  | 257 | 0.33 | 0.38 | 0.10 | 0.32 | 0.20 | 0.34 | 0.17 | 0.29 |  |  |  |  |  |  |
|  | 259 |  | 0.03 |  |  |  |  |  |  |  |  |  |  |  |  |
|  | 261 |  | 0.22 |  | 0.05 | 0.20 | 0.03 |  |  |  |  |  |  |  |  |
|  | 263 |  |  | 0.05 |  |  |  |  |  |  |  |  |  |  |  |
| Me16 | 232 |  |  |  |  |  |  | 0.50 |  |  |  |  |  |  |  |
|  | 236 | 0.79 | 0.56 | 0.35 | 0.27 | 0.35 | 0.35 |  | 0.14 |  |  |  |  |  |  |
|  | 238 |  | 0.28 |  |  |  |  | 0.13 |  |  |  | 0.03 |  |  |  |
|  | 240 |  |  |  |  |  |  |  |  | 0.11 | 0.07 | 0.40 |  |  |  |
|  | 242 |  |  |  |  |  |  | 0.29 |  |  | 0.02 |  |  |  |  |
|  | 244 |  |  |  |  |  |  | 0.08 |  | 0.29 | 0.33 | 0.40 |  |  |  |
|  | 246 |  |  |  |  |  |  |  | 0.07 | 0.61 | 0.36 | 0.05 | 0.97 |  |  |
|  | 248 |  |  |  | 0.14 | 0.10 | 0.03 |  | 0.36 |  | 0.17 | 0.13 | 0.02 |  | 0.13 |
|  | 250 | 0.04 |  |  |  |  |  |  | 0.07 |  | 0.05 |  | 0.02 | 0.06 | 0.06 |
|  | 252 | 0.17 | 0.13 | 0.40 | 0.09 |  | 0.18 |  | 0.14 |  |  |  |  | 0.94 | 0.81 |
|  | 254 |  |  | 0.25 | 0.50 | 0.55 | 0.42 |  | 0.14 |  |  |  |  |  |  |
|  | 256 |  | 0.03 |  |  |  | 0.03 |  |  |  |  |  |  |  |  |
|  | 258 |  |  |  |  |  |  |  | 0.07 |  |  |  |  |  |  |
| Me17 | 121 |  |  |  |  |  |  |  |  | 0.03 |  |  |  |  |  |
|  | 129 |  |  |  |  |  |  |  |  |  |  |  |  | 0.06 |  |
|  | 131 | 0.10 |  | 0.10 |  | 0.10 |  |  |  |  |  |  |  | 0.25 |  |
|  | 133 |  |  | 0.05 | 0.55 |  | 0.30 |  | 0.07 |  |  |  |  |  | 0.14 |
|  | 135 |  |  |  |  |  |  |  | 0.21 |  |  |  |  |  |  |
|  | 137 |  |  |  |  |  |  |  | 0.21 | 0.25 | 0.22 | 0.25 | 0.85 | 0.06 |  |
|  | 139 |  |  |  |  |  |  |  | 0.07 | 0.63 | 0.52 | 0.53 | 0.13 |  |  |
|  | 141 | 0.60 | 0.20 |  |  | 0.20 | 0.09 |  |  |  |  |  |  |  |  |
|  | 145 |  |  |  |  | 0.05 |  |  |  |  |  |  |  |  |  |
|  | 147 | 0.05 | 0.07 |  |  | 0.05 |  |  |  | 0.03 | 0.09 | 0.03 | 0.02 |  |  |
|  | 149 | 0.15 | 0.67 | 0.10 | 0.27 | 0.10 | 0.13 | 0.75 | 0.14 | 0.08 | 0.10 | 0.20 |  |  |  |
|  | 151 |  |  | 0.15 | 0.05 |  |  |  | 0.29 |  |  |  |  |  | 0.43 |
|  | 153 |  |  | 0.25 | 0.05 | 0.25 | 0.32 | 0.17 |  |  |  |  |  | 0.63 | 0.36 |
|  | 155 |  |  | 0.35 | 0.09 |  | 0.15 |  |  |  |  |  |  |  | 0.07 |
|  | 157 | 0.10 |  |  |  |  | 0.01 |  |  |  |  |  |  |  |  |
|  | 159 |  | 0.07 |  |  | 0.25 |  |  |  |  | 0.07 |  |  |  |  |
|  | 163 |  |  |  |  |  |  | 0.08 |  |  |  |  |  |  |  |
